# Supplementary material for: Direct Gαq Gating Is the Sole Mechanism for TRPM8 Inhibition Caused by Bradykinin Receptor Activation
Source: Cell Rep. 2019 Jun 18;27(12):3672–3683.e4. doi: 10.1016/j.celrep.2019.05.080 (PMC6595177; doi:10.1016/j.celrep.2019.05.080)
Supplement: Document S1. Figures S1–S4 [file mmc1.pdf]

Cell Reports, Volume *27*

Supplemental Information

Direct Gα_q Gating Is the Sole Mechanism for TRPM8 Inhibition Caused by Bradykinin Receptor Activation

Xuming Zhang

Supplemental Information

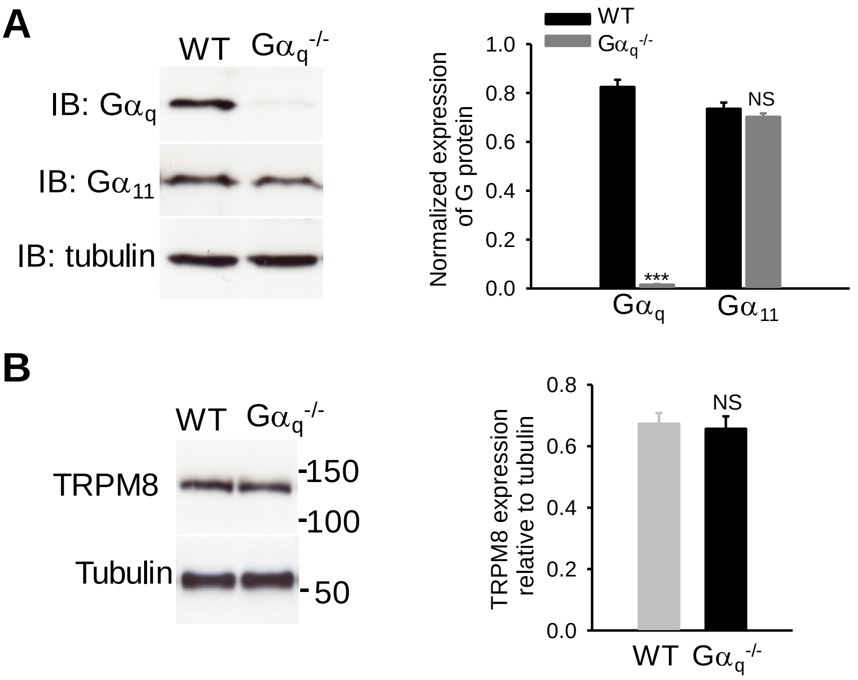

Figure S1. Gα_q is Deleted in DRG neurons from G$\alpha_q^{-/-}$ Mice (related to Figure 1).
(A) Expression of Gα_q and Gα_{11} in DRG neurons from wild-type (WT) and G$\alpha_q^{-/-}$ mice. On the right is the summary of expression of Gα_q and Gα_{11} relative to tubulin from experiments similar to those on the left. *** *P*<0.001; NS, not significant (n=3). **(B)** Example of TRPM8 expression in DRG neurons from WT and G$\alpha_q^{-/-}$ mice. On the right is the quantification of TRPM8 expression relative to that of β-tubulin from experiments similar to those on the left. NS, not significant (n=3).

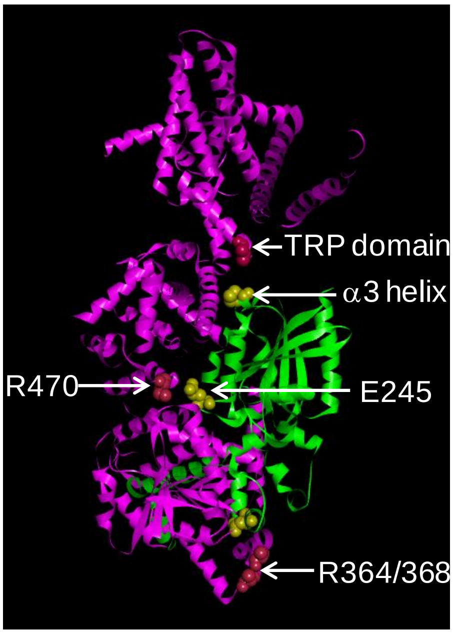

Figure S2. Ribbon Representation of Modelling Interactions between TRPM8 and Gα_q (related to Figure 5). The top TRP domain (V992), lateral R470 and bottom R364/R368 lining the cytoplasmic cavity of TRPM8 are in close proximity to α3 helix (P262), the Switch III domain (E245) and the helical domain in Gα_q (D138),

respectively. Potential interacting residues (R470-E245, R364/368-D138) between these domains are shown as balls.

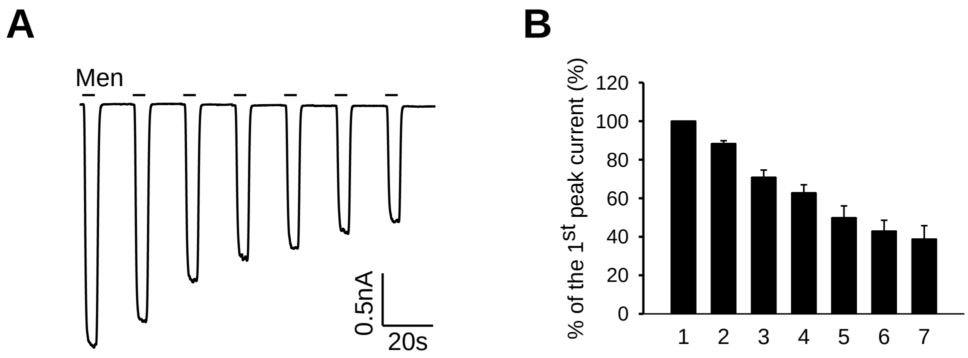

Figure S3. Run-down of TRPM8 Currents (related to Figure 6). (A) Example current traces in HEK293 cells expressing TRPM8 elicited by a series of menthol (200μM, 5s) in Ca^{2+}-free solution. **(B)** Summary of percentage of TRPM8 currents relative to the 1st peak current (n=5).

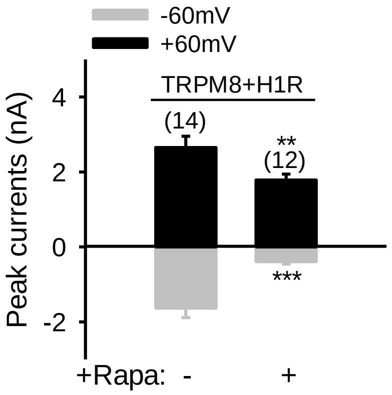

Figure S4. PIP_2 Depletion Inhibits TRPM8 in the Presence of H1R (related to Figure 7). Bar summary of peak inward and outward currents (-60mV and +60mV holding, respectively) of TRPM8 co-expressed with the inducible PIP_2 depletion system and H1R from experiments similar to those in Figure 7C. Depletion of PIP_2 by adding rapamycin (+) (Rapa, 1μM, 1min) inhibited TRPM8 currents. The number of experiments is shown above each bar. **$P < 0.01$; ***$P < 0.001$.
