# Supplementary material for: Direct Gαq Gating Is the Sole Mechanism for TRPM8 Inhibition Caused by Bradykinin Receptor Activation
Source: Cell Rep. 2019 Jun 18;27(12):3672–3683.e4. doi: 10.1016/j.celrep.2019.05.080 (PMC6595177; doi:10.1016/j.celrep.2019.05.080)
Supplement: Document S2. Article plus Supplemental Information [file mmc2.pdf]

Cell Reports

Article

Direct Gα_q Gating Is the Sole Mechanism for TRPM8 Inhibition Caused by Bradykinin Receptor Activation

Graphical Abstract

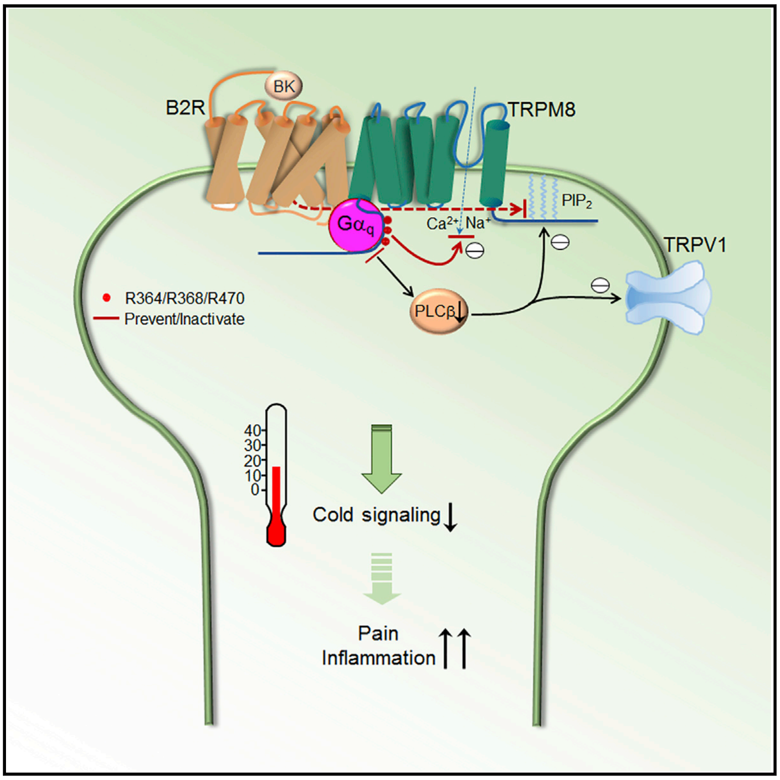

Highlights

- Gα_q governs the cold sensitivity of TRPM8 and TRPM8 inhibition by bradykinin
- Gα_q gates TRPM8 through engaging three basic arginine sites on TRPM8
- TRPM8 inhibition by bradykinin depends on Gα_q gating sites but not PIP_2 signaling
- Bradykinin receptor prevents the PIP_2 sensitivity of TRPM8 channels

Authors

Xuming Zhang

Correspondence

x.zhang39@aston.ac.uk

In Brief

TRPM8 channels are inhibited by receptors coupled to Gα_q, contributing to pain and inflammation. Zhang reveals Gα_q gating sites on TRPM8 and shows that bradykinin receptor solely uses Gα_q gating sites for TRPM8 inhibition upon activation, while depriving the channel of sensitivity to PIP_2.

Zhang, 2019, Cell Reports 27, 3672–3683
June 18, 2019 Crown Copyright © 2019
https://doi.org/10.1016/j.celrep.2019.05.080

OPEN ACCESS
CellPress

Cell Reports
Article

Direct $G\alpha_q$ Gating Is the Sole Mechanism for TRPM8 Inhibition Caused by Bradykinin Receptor Activation

Xuming Zhang[1,2,*]
[1]School of Life and Health Sciences, Aston University, Aston Triangle, Birmingham B4 7ET, UK
[2]Lead Contact
*Correspondence: x.zhang39@aston.ac.uk
https://doi.org/10.1016/j.celrep.2019.05.080

SUMMARY

Activation of $G\alpha_q$-coupled receptors by inflammatory mediators inhibits cold-sensing TRPM8 channels, aggravating pain and inflammation. Both $G\alpha_q$ and the downstream hydrolysis of phosphatidylinositol 4, 5-bisphosphate (PIP_2) inhibit TRPM8. Here, I demonstrate that direct $G\alpha_q$ gating is essential for both the basal cold sensitivity of TRPM8 and TRPM8 inhibition elicited by bradykinin in sensory neurons. The action of $G\alpha_q$ depends on binding to three arginine residues in the N terminus of TRPM8. Neutralization of these residues markedly increased sensitivity of the channel to agonist and membrane voltage and completely abolished TRPM8 inhibition by both $G\alpha_q$ and bradykinin while sparing the channel sensitivity to PIP_2. Interestingly, the bradykinin receptor B2R also binds to TRPM8, rendering TRPM8 insensitive to PIP_2 depletion. Furthermore, TRPM8-$G\alpha_q$ binding impaired $G\alpha_q$ coupling and signaling to PLCβ-PIP_2. The crosstalk in the TRPM8-$G\alpha_q$-B2R complex thus determines $G\alpha_q$ gating rather than PIP_2 as a sole means of TRPM8 inhibition by bradykinin.

INTRODUCTION

TRPM8 channels detect a wide range of cold temperatures, spanning from innocuous cooling to noxious cold (Madrid et al., 2009; McKemy et al., 2002; Peier et al., 2002). It is also activated by cooling-mimetic compounds, such as menthol and its derivative, WS-12. The basal cold sensitivity of TRPM8 is critical to many physiological processes ranging from cold sensation to core body temperature regulation (Bautista et al., 2007; Colburn et al., 2007; Dhaka et al., 2007; Gavva et al., 2012), to basal tear secretion (Parra et al., 2010). Furthermore, activation of TRPM8 inhibits inflammatory and neuropathic pain, mediating an analgesic effect (Dhaka et al., 2007; Knowlton et al., 2013; Liu et al., 2013; Proudfoot et al., 2006), though it causes cold hypersensitivity in some cases (Colburn et al., 2007; De Caro et al., 2018; Knowlton et al., 2013). TRPM8 has also been implicated in the inhibition of tissue inflammatory responses and inflammatory cytokine release (Caceres et al., 2017; Ramachandran et al., 2013; Wang et al., 2017). Activation of TRPM8, therefore, inhibits both pain and inflammation, which may underlie cold therapy that has been used to relieve pain and inflammation for hundreds of years.

However, TRPM8 was inhibited during inflammation due to the actions of released inflammatory mediators such as bradykinin (BK) and histamine (Linte et al., 2007; Premkumar et al., 2005; Zhang et al., 2012). Inhibition of TRPM8, therefore, disinhibits the anti-pain and anti-inflammatory effects of TRPM8, contributing to aggravated pain, inflammation, and possibly dry eye diseases (Liu et al., 2013; Yang et al., 2018; Zhang et al., 2012).

BK and histamine act on $G\alpha_q$-coupled receptors. Initially, TRPM8 inhibition induced by BK was attributed to dephosphorylation of TRPM8 by activated protein kinase C (PKC) (Premkumar et al., 2005). Another appealing possibility is that activated $G\alpha_q$-PLCβ catalyzes hydrolysis of the membrane lipid phosphatidylinositol 4, 5-bisphosphate (PIP_2), which is essential for TRPM8 activation (Liu and Qin, 2005; Rohács et al., 2005), leading to TRPM8 inhibition. Indeed, PIP_2 hydrolysis-mediated TRPM8 inactivation has been implicated in TRPM8 desensitization and cold adaptation (Daniels et al., 2009; Rohács et al., 2005).

Adding to the complexity, we have found that activated $G\alpha_q$ directly inhibits TRPM8 *in vitro* (Zhang et al., 2012). However, it is not yet clear whether direct $G\alpha_q$ gating of TRPM8 also occurs in native sensory neurons and which mechanisms are mainly responsible for TRPM8 inhibition by inflammatory mediators *in vivo*. After all, activated $G\alpha_q$ will inevitably activate downstream PLCβ, triggering the concomitant breakdown of PIP_2. It is also not known how $G\alpha_q$ directly gates TRPM8 and the relative role of $G\alpha_q$ gating and PIP_2 signaling in TRPM8 modulation.

In this research, I have found that direct $G\alpha_q$ gating is crucial for regulating the basal cold sensitivity of TRPM8 and is the sole mechanism of TRPM8 inhibition elicited by BK in native sensory neurons without significant involvement of PLCβ-PIP_2 signaling or $G\alpha_{11}$ in this process. Importantly, I revealed the $G\alpha_q$ gating mechanism and identified three basic arginine residues on the N-terminal melastatin homology region (MHR) 1–3 in TRPM8 as $G\alpha_q$ gating sites. Mutation of these sites entirely abolished TRPM8 inhibition by activated $G\alpha_q$ and BK and also significantly reduced TRPM8 inhibition by histamine. I further elucidated the mechanism for the lack of a role of PIP_2 in TRPM8 inhibition by BK, and I found that it is determined by a bidirectional crosstalk in the TRPM8-$G\alpha_q$-B2R complex. Our data also suggest independent and cooperative modulation of TRPM8 by $G\alpha_q$ and PIP_2.

 Crown Copyright © 2019
This is an open access article under the CC BY license (http://creativecommons.org/licenses/by/4.0/).

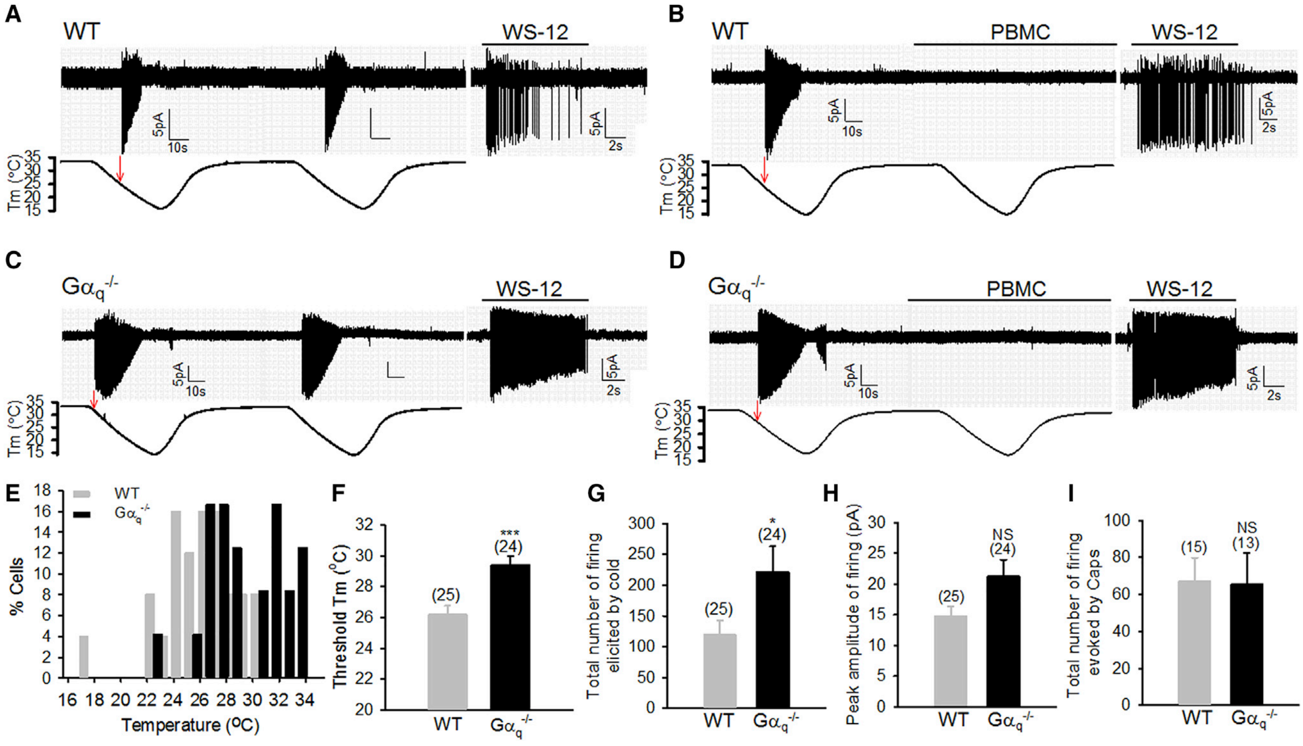

Figure 1. $G\alpha_q$ Is Crucial for the Cold Sensitivity of TRPM8[+] DRG Neurons

(A–D) Firing responses evoked by two consecutive cold ramps and WS-12 (1 μM) in wild-type (WT) (A and B) and $G\alpha_q^{-/-}$ (C and D) DRG neurons in the absence (A and C) or presence (B and D) of PBMC (50 nM). Arrows indicate cold activation threshold.

(E) Histogram distribution of cold activation threshold of DRG neurons from experiments similar to those in (A)–(D). WT, n = 25; $G\alpha_q^{-/-}$, n = 24.

(F) Bar summary of threshold for cold activation of DRG neurons.

(G and H) Summary of total number of firing (G) and average peak amplitude of firing (H) responses evoked during cold application period from the same experiments as described above.

(I) Summary of total number of firing responses evoked during capsaicin application (0.5 μM, 10 s).

Error bars in all figures represent mean ± SEM. *p < 0.05; ***p < 0.001; NS, not significant. See also Figure S1.

RESULTS

$G\alpha_q$ Is Crucial for the Cold Sensitivity of TRPM8 in Sensory Neurons

We have previously shown that activated $G\alpha_q$ directly inhibits TRPM8 channels *in vitro* (Zhang et al., 2012), but it remains unknown whether this mechanism also occurs to sensory neurons. To discriminate between $G\alpha_q$ gating and PIP_2 signaling in TRPM8 modulation in sensory neurons, I took advantage of $G\alpha_q$ knockout (KO) mice, in which $G\alpha_{11}$ remains intact and will take over the activation of downstream PLCβ-PIP_2 signaling.

Cell-attached patch clamping was used to record TRPM8-mediated firing responses in small-to-medium sensory dorsal root ganglia (DRG) neurons, because the cell-attached mode preserves intracellular modulatory factors, minimizing artificial disruption of intracellular signaling (Madrid et al., 2006); furthermore, TRPM8 exhibits run-down in other patch configurations (Liu and Qin, 2005). Figure 1A shows that a ramp drop in bath temperatures elicited firing discharges in a DRG neuron at 26.6°C. A second cold ramp evoked similar firing responses with little desensitization. To verify that cold-elicited firing is mediated by TRPM8, DRG neurons were exposed to PBMC, a specific TRPM8 antagonist (Knowlton et al., 2011), during the second cold ramp. As shown in Figure 1B, PBMC completely blocked firing induced by the second cold challenge. Furthermore, the same neurons also responded to the specific TRPM8 agonist WS-12 (Figures 1A and 1B), validating that cold-induced firing is mediated by TRPM8. The effects of PBMC and WS-12 were, therefore, used to identify TRPM8-mediated cold responses in DRG neurons. With this approach, ~30% of probed cells responded to cold, and ~68% of these cold-sensitive neurons were TRPM8[+]. TRPM8-independent firing responses were excluded from further analysis.

I then performed similar experiments in DRG neurons isolated from $G\alpha_q$-deficient mice. I first validated the ablation of $G\alpha_q$, but not $G\alpha_{11}$, in $G\alpha_q$-lacking DRG neurons (Figure S1A). $G\alpha_q$-deficient DRG neurons responded to two consecutive cold ramps similarly to wild-type (WT) neurons (Figure 1C), but they started to fire at a higher temperature threshold (Figures 1C and 1D). The cold responses were blocked by PBMC, and the same neurons also responded to WS-12, confirming that cold-evoked firing responses are TRPM8 dependent (Figures 1C and 1D).

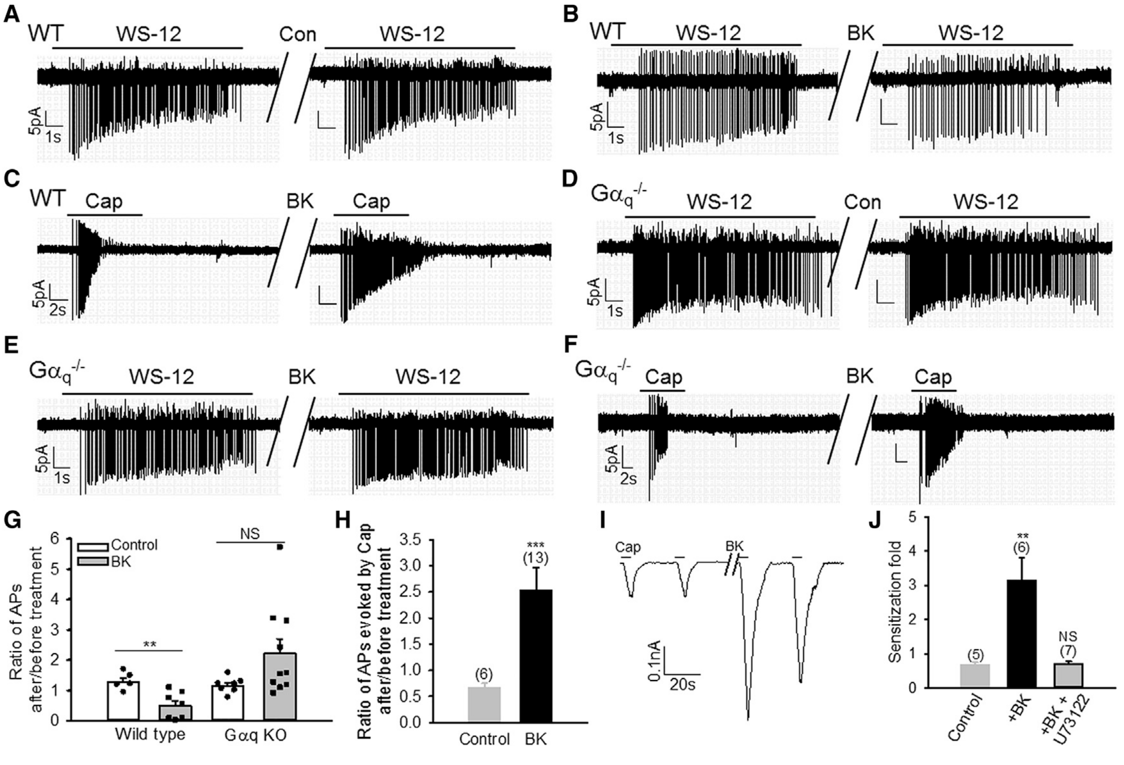

Figure 2. $G\alpha_q$ Is Essential for BK-Induced Inhibition of TRPM8-Dependent Firing Responses in DRG Neurons

(A–C) Representative firing responses elicited by WS-12 (1 μM) or capsaicin (Cap, 0.5 μM) (C) in WT DRG neurons before and after vehicle (A) or BK (1 μM, 1 min) (B and C). (B) and (C) indicate the same neuron.

(D–F) Example firing responses evoked by WS-12 or capsaicin (F) in DRG neurons lacking $G\alpha_q$ before and after vehicle (D) or BK (E and F). (E) and (F) indicate the same neuron.

(G) Scatterplot of ratio of action potentials (APs) induced by WS-12 after and before BK in experiments similar to those in (A)–(D). **p = 0.00588; NS, p = 0.107.

(H) Summary of ratio of firing responses evoked by capsaicin after and before BK from experiments similar to those in (C) and (F).

(I) Example currents evoked by capsaicin (100 nM, 5 s) in a $G\alpha_q$ null DRG neuron. The gap indicates BK treatment (1 μM, 1 min).

(J) Collective results of sensitization fold caused by BK from experiments similar to those in (I). The sensitization was abolished by U73122 (2.5 μM). The number of experiments is denoted above each bar.

All data indicate mean ± SEM. **p < 0.01; ***p < 0.001; NS, not significant.

Overall, deleting $G\alpha_q$ markedly shifted the temperature threshold for TRPM8 activation in DRG neurons toward higher temperature ranges in both low- and high-threshold cold-sensing neurons (Figure 1E), leading to a significant increase of 3.2°C in the cold activation threshold (Figure 1F) (WT, 26.16°C ± 0.6°C; $G\alpha_q^{-/-}$, 29.37°C ± 0.61°C; p < 0.001), consistent with the finding of the basal inhibition of TRPM8 channels by endogenous $G\alpha_q$ (Zhang et al., 2012). Notably, cold temperatures also induced much more TRPM8-dependent firing events in $G\alpha_q$ KO neurons than in WT neurons (Figures 1A–1D and 1G), though there was no significant difference in firing amplitude between WT and KO neurons (Figure 1H). Enhanced TRPM8 responses are not caused by indirect upregulation of TRPM8, because TRPM8 expression was not altered in $G\alpha_q$ KO neurons (Figure S1B). In contrast to cold, firing events induced by capsaicin were not significantly different between WT and $G\alpha_q$ KO neurons (Figure 1I), suggesting that increased firing responses in $G\alpha_q$-lacking neurons are specific to cold and unlikely to be mediated by indirect effects of $G\alpha_q$ deletion on other action potential transducing channels such as voltage-gated sodium channels.

These experiments demonstrate that $G\alpha_q$ tonically inhibits the basal cold sensitivity of TRPM8 channels in DRG neurons and suggest that $G\alpha_q$ is present in both low- and high-threshold cold-sensing DRG neurons.

$G\alpha_q$ Is Essential for Inhibiting Firing Responses Induced by Bradykinin

To investigate whether $G\alpha_q$ is also crucial for TRPM8 inhibition induced by BK in DRG neurons, cells were stimulated by WS-12, and induced firing activity was monitored with cell-attached recording. Under control conditions, no significant changes in firing were observed in either WT or $G\alpha_q$ KO neurons after vehicle perfusion (1 min) (Figures 2A, 2D, and 2G). The same procedure was then used to investigate the effect of BK on TRPM8. Of note, the functional BK receptor B2R is co-expressed in 33.3% $TRPM8^+$ DRG neurons (Zhang et al., 2012) but in 83.9% $TRPV1^+$ DRG neurons, indicated by the proportion of neurons in which TRPV1 is sensitized by BK (Zhang et al., 2008) (also discussed later). In fact, BK-induced inhibition of TRPM8 was only observed in $TRPV1^+$ neurons (Zhang et al.,

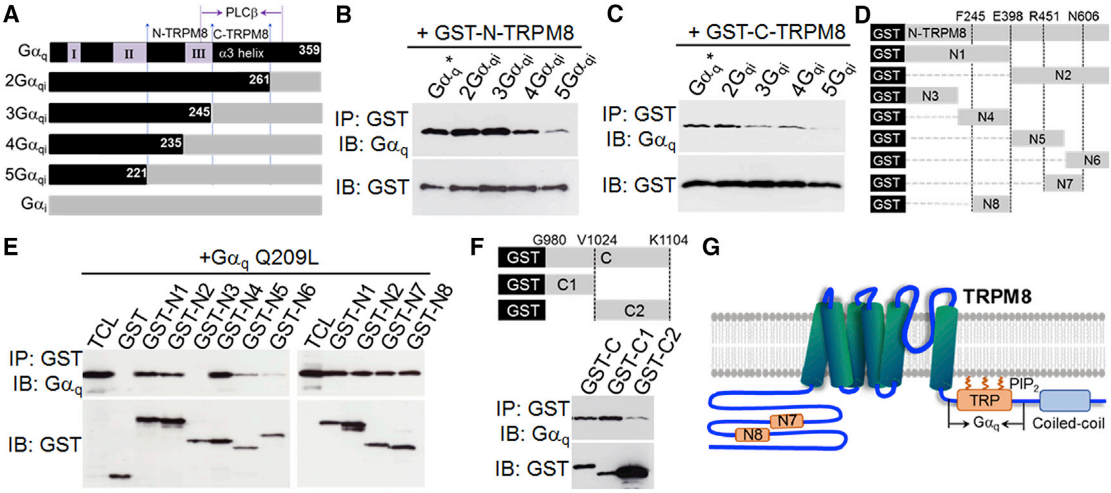

Figure 3. Delineation of $G\alpha_q$ Binding Regions in TRPM8

(A) Schematic diagram shows different $G\alpha_{qi}$ chimeras and regions binding to the N- and C-terminus of TRPM8, and PLCβ.

(B and C) GST-coupled N terminus (B) and C terminus (C) of TRPM8 bind to different $G\alpha_q$ chimeras in pull-down assays. IP, immunoprecipitation.

(D) Schematic diagram shows different N-terminal fragments coupled to GST.

(E) The binding of $G\alpha_q$ Q209L to different GST-coupled N-terminal fragments of TRPM8. Total cell lysate (TCL) denotes $G\alpha_q$ expression.

(F) The binding of $G\alpha_q$ Q209L to different C-terminal fragments coupled to GST (top) in a GST pull-down assay (bottom).

(G) Schematic diagram depicts the relationship of $G\alpha_q$ binding regions (N7, N8, and TRP) and other domains.

2012). Therefore, to ensure that the recorded neurons express B2R and to preclude the neurons lacking B2R from confounding analysis, I selected TRPM8$^+$ DRG neurons exhibiting TRPV1 sensitization and/or TRPM8 inhibition in response to BK as an index of B2R expression. As expected, WS-12-elicited firing responses were significantly inhibited and even completely eliminated by BK in some WT DRG neurons (Figures 2B and 2G). Furthermore, the response to capsaicin in this neuron was also sensitized by BK (Figure 2C), confirming functional B2R expression. In contrast, BK did not inhibit TRPM8-triggered firing in $G\alpha_q$-lacking DRG neurons and, in fact, slightly increased firing elicited by WS-12, though it was not statistically significant compared to control (Figures 2E and 2G). The lack of inhibitory effect was not due to lack of expression of B2R or $G\alpha_{11}$ or impaired PLCβ signaling in $G\alpha_q$-deficient DRG neurons, because firing responses elicited by capsaicin in the same neurons were sensitized by BK (Figures 2F and 2H), an event depending on the $G\alpha_{q/11}$-PLCβ signaling pathway (Lukacs et al., 2007; Zhang et al., 2008); furthermore, $G\alpha_{11}$ is expressed in every DRG neuron (Han et al., 2006). As complementary evidence, BK also sensitized TRPV1 in $G\alpha_q$ null neurons to a degree similar to that in WT neurons in whole-cell patch clamping (Figures 2I, 2J, and 7J); moreover, the sensitization was completely prevented by the phospholipase C (PLC) inhibitor U73122, validating the involvement of PLCβ in this process. These data suggest that PLCβ signaling is not impaired in DRG neurons lacking $G\alpha_q$, consistent with the finding that $G\alpha_q$, rather than $G\alpha_{11}$, plays a major role in TRPM8 inhibition (Li and Zhang, 2013; Zhang et al., 2012).

Altogether, these experiments demonstrate that $G\alpha_q$ is crucial for both the basal and BK-induced TRPM8 inhibition in DRG neurons. Neither $G\alpha_{11}$ nor downstream PLCβ signaling is involved in these processes.

Identification of $G\alpha_q$ Effector Sites on TRPM8 Channels

Activated $G\alpha_q$ rapidly inhibits TRPM8 through binding to TRPM8 (Zhang et al., 2012). Therefore, $G\alpha_q$ binding sites on TRPM8 should constitute critical channel gating sites. To ascertain which terminus of TRPM8 contains functional $G\alpha_q$ binding sites, the GST (glutathione S-transferase)-coupled N or C terminus of TRPM8 was used to pull down different chimeric $G\alpha_q$ proteins in which different functional regions in the distal C terminus of $G\alpha_q$ are replaced by $G\alpha_i$ (Figure 3A) (Zhang et al., 2012). In general, the binding of $G\alpha_q$ to the N terminus is stronger than that to the C terminus of TRPM8 (Figures 3B and 3C), suggesting that the N terminus of TRPM8 is more critical to $G\alpha_q$ gating. The most potent binding to the N terminus of TRPM8 was found with $G\alpha_{qi}$, $2G\alpha_{qi}$, and $3G\alpha_{qi}$ (Figure 3B), which also exert a potent inhibitory effect on TRPM8 (Zhang et al., 2012). Reduced binding to the N terminus was observed with $4G\alpha_{qi}$ and $5G\alpha_{qi}$ (Figure 3B), which exhibit a diminished inhibitory effect on TRPM8 due to the replacement of Switch III in $G\alpha_q$ by the equivalent part from $G\alpha_i$ in these chimeras (Figure 3A) (Zhang et al., 2012). The good correlation between the binding of $G\alpha_q$ chimeras to the N terminus and the functional effect on TRPM8 further suggests that the N terminus of TRPM8 contains $G\alpha_q$ gating sites. The binding experiment also supports that the N terminus mainly binds to Switch III in $G\alpha_q$ (Figure 3A), a major region also responsible for the gating of TRPM8 (Zhang et al., 2012), consistent with the suggestion of a critical role of the N-terminal TRPM8 in $G\alpha_q$ gating. In contrast, reduced binding to the C terminus of TRPM8 was observed in $3G\alpha_{qi}$ (Figure 3C), in which the α3 helix downstream of Switch III in $G\alpha_q$ is replaced (Figure 3A), suggesting that the C terminus of TRPM8 primarily binds to the α3 helix, a region also responsible for binding to PLCβ (Figure 3A) (Venkatakrishnan and Exton, 1996; Waldo et al., 2010).

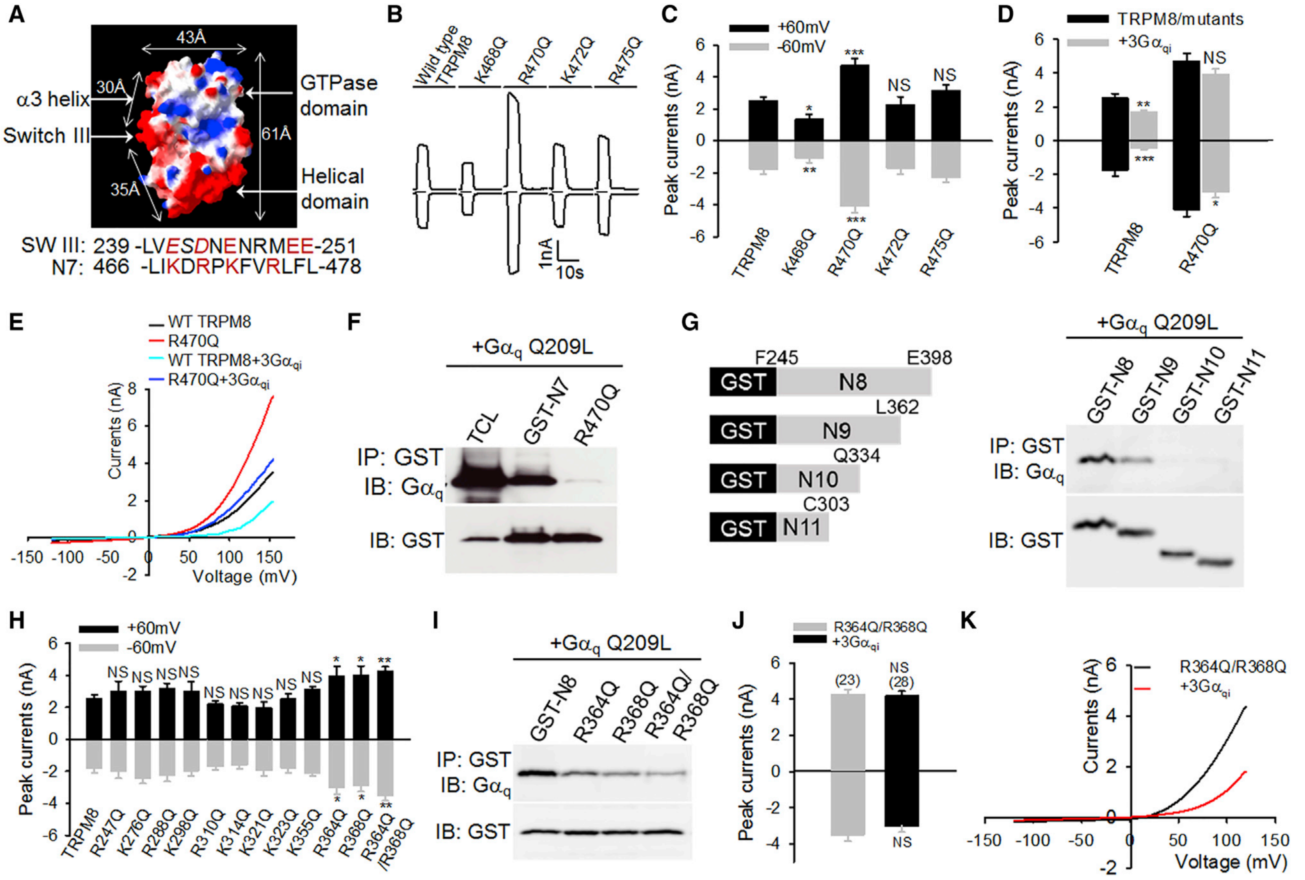

Figure 4. Identification of $G\alpha_q$ Effector Sites on TRPM8

(A) Molecular surface charge representation of $G\alpha_q$. Key domains and distance are shown. Underneath is the alignment of charged residues (in red) in Switch III of $G\alpha_q$ and N7-TRPM8.

(B) Example inward and outward whole-cell currents elicited by menthol (200 μM, 5 s) at −60 mV and 60 mV, respectively, in HEK293 cells expressing TRPM8 and mutants as indicated.

(C) Summary of TRPM8 peak currents from similar experiments to those in B (n = 19–28).

(D) Summary of the effect of $3G\alpha_{qi}$ on the peak currents of TRPM8 and R470Q elicited by menthol (n = 22–35).

(E) Averaged current-voltage (I-V) traces of TRPM8 and R470Q with or without $3G\alpha_{qi}$ co-expression (n = 16–27).

(F) Binding of $G\alpha_q$ Q209L to GST-N7 and R470Q mutant in a GST pull-down assay.

(G) On the left is a diagram showing different truncations of N8-TRPM8. On the right, the binding of truncated N8 fragments to $G\alpha_q$ Q209L is indicated.

(H) Summary of inward and outward currents of different TRPM8 mutants elicited by menthol.

(I) GST pull-down assay shows the binding of $G\alpha_q$ Q209L to GST-N8 and mutants as indicated.

(J) Summary of currents of R364Q-R368Q mutant with or without co-expression of $3G\alpha_{qi}$. The number of experiments is indicated above each bar.

(K) Average I-V relationship of R364Q-R368Q mutant with (n = 27) or without (n = 20) the co-presence of $3G\alpha_{qi}$.

All error bars represent mean ± SEM. *p < 0.05; **p < 0.01; ***p < 0.001; NS, not significant.

To further delineate $G\alpha_q$ binding regions in the N terminus, a series of different N-terminal fragments coupled to the GST tag were constructed for $G\alpha_q$ pull-down assay (Figure 3D). With this approach, two small regions, N7 (F245-E398) and N8 (R451-N606), were found to be responsible for the binding of $G\alpha_q$ to the N terminus (Figures 3E and 3G). These two short regions, therefore, likely contain the functional $G\alpha_q$ effector sites. Using a similar approach, I have also found that the main $G\alpha_q$ binding region in the C terminus is located in the proximal C terminus of TRPM8 (Figure 3F), a region also containing the TRP domain critical to TRPM8 gating and modulation (Figure 3G). Taken together, $G\alpha_q$ binds to N7 and N8 in the N terminus and also to the proximal C terminus of TRPM8, although N7 and N8 are more likely to be the functional $G\alpha_q$ gating regions (Figure 3G). Reciprocally, the N and C termini of TRPM8 mainly bind to Switch III and the α3 helix in $G\alpha_q$, respectively (Figure 3A).

Activated $G\alpha_q$ gates TRPM8 through Switch III (Zhang et al., 2012), a region undergoing major conformational changes upon exchange of guanosine triphosphate (GTP) for guanosine diphosphate (GDP) on $G\alpha_q$ during $G\alpha_q$ activation (Lambright et al., 1996; Waldo et al., 2010), consistent with the structural requirement for effector interaction. Notably, Switch III contains a cluster of acidic residues. Surface charge modeling of $G\alpha_q$ also revealed that Switch III is negatively charged (Figure 4A). Therefore, I

hypothesized that the $G\alpha_q$ effector sites on TRPM8 are positive, allowing for electrostatic interaction with negative Switch III in $G\alpha_q$. Interestingly, a cluster of basic arginine and lysine residues were found on N7, with a distribution pattern similar to those of acidic residues on Switch III in $G\alpha_q$ (Figure 4A). These basic residues were then neutralized by mutation to glutamine.

In principle, the mutation of $G\alpha_q$ effector sites on TRPM8 should disrupt $G\alpha_q$ binding, abolish the inhibitory effect of $G\alpha_q$, and potentiate the function of TRPM8 channels. Indeed, I found that neutralizing R470 markedly enhanced TRPM8 inward and outward currents evoked by menthol, while mutation of other nearby basic residues either reduced TRPM8 currents or had no significant effect (Figures 4B and 4C). Moreover, R470Q mutant abolished the inhibition of outward current and reduced the inhibition of inward current caused by $3G\alpha_{qi}$, an activated $G\alpha_q$ chimera containing Q209L mutation, while downstream signaling to PLCβ was selectively ablated (Figure 4D) (Zhang et al., 2012), although $3G\alpha_{qi}$ still significantly inhibited the inward current of R470Q (Figure 4D). Depolarization-induced outward currents in R470Q were also much larger than those in WT TRPM8 but were still inhibited by $3G\alpha_{qi}$, as seen in the WT channel (Figure 4E). Notably, mutating R470 completely eliminated the binding of activated $G\alpha_q$ to the N7 fragment (Figure 4F), demonstrating that R470 is crucial to the binding between $G\alpha_q$ and N7. Taken together, these results suggest that R470 is a critical $G\alpha_q$ effector site on TRPM8. However, the incomplete abrogation of $G\alpha_q$-elicited inhibition of TRPM8 by R470Q mutation suggests the presence of other unidentified $G\alpha_q$ effector sites, likely on N8 (discussed later).

There are no obvious clusters of positive residues on N8. To narrow down the $G\alpha_q$ binding region in N8, a progressive truncation of N8 coupled to GST was generated and used to pull down $G\alpha_q$ (Figure 4G). A prominent reduction in $G\alpha_q$ binding was observed when the distal 36 amino acids were truncated from the C terminus of N8 (Figure 4G), suggesting that these 36 amino acids contain $G\alpha_q$ binding sites. Meanwhile, basic residues in N8 were individually neutralized to glutamine. Currents mediated by these mutated channels were then monitored using whole-cell patch clamping. I found that neutralization of two individual arginine residues (R364Q and R368Q) on N8 both significantly enhanced TRPM8 currents (Figure 4H). TRPM8 currents were further increased after mutating these two sites in combination (R364Q-R368Q) (Figure 4H). However, all other mutants were without significant effect. Coincidently, R364 and R368 fall within the distal 36 amino acids in N8 that are also critical to $G\alpha_q$ binding (Figure 4G). As anticipated, neutralization of these two sites in N8 was also sufficient to disrupt the binding between N8 and $G\alpha_q$ (Figure 4I), suggesting that these two sites are also $G\alpha_q$ effector sites. Consistent with this idea, the inhibitory effects of $3G\alpha_{qi}$ on the inward and outward currents of TRPM8 evoked by menthol were abolished in the double mutant (R364Q-R368Q) (Figure 4J). However, voltage gating of R364Q-R368Q was still inhibited by $3G\alpha_{qi}$ (Figure 4K).

Altogether, $G\alpha_q$ binds to two small regions in the N terminus of TRPM8 (N7 and N8), and three positively charged arginine residues (R364, R368, and R470) within these regions function as $G\alpha_q$ effector sites. These experiments also support the notion that gating of TRPM8 by menthol and voltage is separable through independent mechanisms, though they are allosteric during TRPM8 gating.

$G\alpha_q$ Effector Sites Are Responsible for $G\alpha_q$ Gating of TRPM8

I next mutated all three basic arginine residues on TRPM8 to glutamine and named the mutant TRPM8-TM (triple mutant). As expected, TRPM8-TM exhibited much larger currents than the WT channel and completely abolished the inhibitory effects of $3G\alpha_{qi}$ on TRPM8 currents elicited by menthol (Figures 5A and 5B). The sensitivity of TRPM8-TM to menthol was also dramatically increased, leading to a marked leftward shift in dose-response curve compared to that of WT TRPM8 (WT, EC_{50} = 148.8 μM ± 8.1 μM; TM, EC_{50} = 40.6 μM ± 5.3 μM) (Figure 5C). However, this marked leftward shift was not observed in mouse embryonic fibroblast (MEF) cells lacking endogenous $G\alpha_{q/11}$ (TRPM8 in $G\alpha_{q/11}{}^{-/-}$, EC_{50} = 75.1 μM ± 2.8 μM; TM in $G\alpha_{q/11}{}^{-/-}$, EC_{50} = 61.4 μM ± 5.0 μM), supporting the idea that increased sensitivity of TRPM8-TM is due to relief of basal inhibition from endogenous $G\alpha_{q/11}$ and not caused by indirect alterations to the intrinsic biophysical properties of the channel. These data are also consistent with the basal inhibition of TRPM8 by endogenous $G\alpha_q$ in DRG neurons (Figures 1C–1G). Notably, TRPM8-TM also entirely abolished the inhibition of $3G\alpha_{qi}$ on TRPM8 outward currents evoked by a depolarization voltage ramp (Figure 5D). A further detailed analysis of voltage gating of TRPM8 through applying a series of depolarization voltage steps revealed that TRPM8-TM shifted the voltage gating of TRPM8 toward negative potentials close to resting membrane potential, resulting in a markedly reduced $V_{1/2}$ (WT, $V_{1/2}$ = 116.0 mV ± 11.0 mV; TM, $V_{1/2}$ = 66.5 mV ± 5.9 mV) (Figures 5E–5G). It also shows that TRPM8-TM abolished the inhibitory effect of activated $3G\alpha_{qi}$ on $V_{1/2}$ observed in the WT channels (Figure 5G) (Zhang et al., 2012).

Altogether, TRPM8-TM completely abolished the inhibitory effects of $3G\alpha_{qi}$ on TRPM8 activation triggered by both menthol and voltage, demonstrating that three arginine residues (R364, R368, and R470) in the N terminus of TRPM8 are $G\alpha_q$ effector sites responsible for the $G\alpha_q$ gating of TRPM8.

In accordance with the recently revealed TRPM8 structure (Yin et al., 2018), R364 and R368 are located near the bottom stack layer of the cytoplasmic domain (CD) of TRPM8, sitting at a turning loop between two helixes in MHR1-2 (Figure 5H). R470 also sits at a loop between two helixes (HTH3a and HTH3b) in the MHR3 domain. Three arginine residues constitute a contiguous plane lining a cytoplasmic cavity formed by the CD of TRPM8 (Figure 5I). Of note, the C-terminal $G\alpha_q$ binding TRP domain and the bottom S6 from the pore domain form the dome of the cytoplasmic cavity. The height of the cavity measured from the bottom of S6 to R368 is 83 Å. The distance of R470 from two opposite subunits is 68 Å, and the distance of two opposite R368 residues is 82 Å (Figure 5I). Therefore, the size of the cavity is, in principle, sufficient to accommodate $G\alpha_q$ with a dimension of 43 Å × 61 Å (Figure 4A). In this case, the top of $G\alpha_q$ would be close to the TRP domain and the intracellular mouth of the channel pore. Conformational changes in $G\alpha_q$ upon activation may be transduced to the TRP domain and the pore domain, resulting in the allosteric gating of TRPM8.

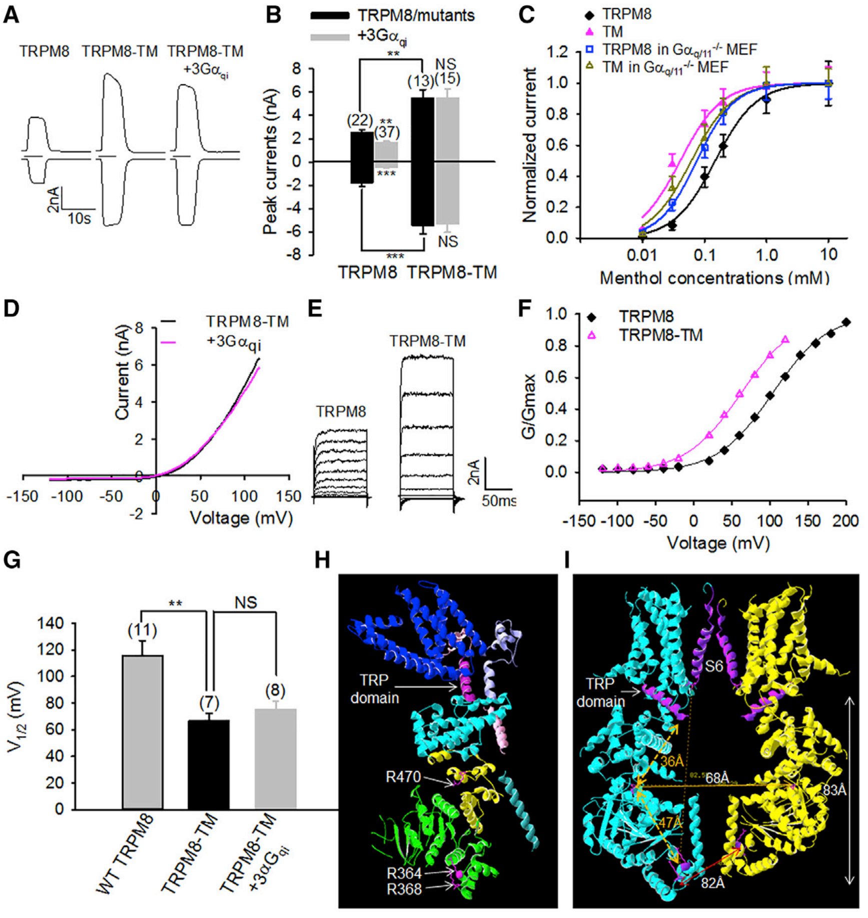

Figure 5. $G\alpha_q$ Gates TRPM8 through Three Arginine Residues on TRPM8

(A) Representative inward and outward currents of TRPM8 and TRPM8-TM activated by menthol (200 μM, 5 s).

(B) Summary of currents of TRPM8 and TRPM8-TM with or without $3G\alpha_{qi}$ from experiments similar to those in (A). Data are mean ± SEM. **p < 0.01; ***p < 0.001.

(C) Dose-response relationship of TRPM8 and TRPM8-TM expressed in HEK293 and $G\alpha_{q/11}^{-/-}$ MEF cells in response to menthol. Curves are fitted with the Hill equation. TRPM8 (◆), EC_{50} = 148.8 μM ± 8.1 μM; TRPM8-TM (▲), EC_{50} = 40.6 μM ± 5.3 μM; TRPM8 in $G\alpha_{q/11}^{-/-}$ MEF (□), EC_{50} = 75.1 μM ± 2.8 μM; TM in $G\alpha_{q/11}^{-/-}$ (△), EC_{50} = 61.4 μM ± 5.0 μM). n = 7–15.

(D) Average I-V relationship of TRPM8-TM with (pink, n = 13) or without (black, n = 14) $3G\alpha_{qi}$.

(E) Example currents of TRPM8 and TRPM8-TM evoked by voltage steps from −120 mV up to 200 mV in 20-mV increments. Maximal current was evoked at 140 mV for TRPM8-TM.

(F) Normalized conductance (G)-voltage relationship from the cells in (E) fitted with the Boltzmann equation, giving rise to $V_{1/2}$ and slope factor as follows: TRPM8, 103.5 mV and 36.2 mV; TRPM8-TM, 62.0 mV and 36.3 mV.

(G) Collective results of $V_{1/2}$ from experiments similar to those in (E) and (F). The number of experiments is shown above each bar.

(H) Side stereoview of a ribbon representation of the TRPM8 structure (6BPQ).

(I) Side stereoview of central cavity formed by the N terminus of two opposite TRPM8 subunits. Distance measurements between domains and residues are indicated.

Error bars represent mean ± SEM. **p < 0.01; NS, not significant. See also Figure S2.

$G\alpha_q$ Gating Is Crucial for TRPM8 Inhibition by Inflammatory Mediators

The inflammatory mediators BK and histamine inhibit TRPM8 through binding, respectively, to B2R and H1R, two $G\alpha_q$-coupled G-protein-coupled receptors (GPCRs). Both activated $G\alpha_q$ and concomitant PIP_2 hydrolysis cause TRPM8 inhibition. The identified TRPM8-TM selectively deficient for $G\alpha_q$ gating but without impairment in PLCβ signaling and PIP_2 sensitivity (discussed later) will be an excellent tool for determination of the relative role of direct $G\alpha_q$ gating and PIP_2 signaling in TRPM8 inhibition during inflammatory signaling.

TRPM8 undergoes run-down in the whole-cell configuration, even under Ca^{2+}-free conditions (Figure S3), likely due to the breakdown of intracellular PIP_2 and/or polyphosphate (Liu and Qin, 2005; Zakharian et al., 2009). Furthermore, intracellular dialysis under the whole-cell configuration may disrupt $G\alpha_q$ coupling and GPCR signaling. To avoid these artificial effects and to minimize the intervention of whole-cell dialysis in intracellular signaling, cells were briefly pretreated with inflammatory mediators (1 min), allowing for intact completion of intracellular signaling before entering the whole-cell configuration for monitoring TRPM8 currents. In HEK293 cells co-expressing TRPM8 and B2R, a short pretreatment with BK elicited a robust inhibition of the inward and outward currents of TRPM8 (Figures 6A and 6B), consistent with the previous findings (Zhang et al., 2012). However, the potent inhibition was completely abolished in TRPM8-TM (Figures 6A and 6B). Similarly, TRPM8-TM also completely abrogated BK-elicited inhibition of voltage gating of TRPM8 (Figure 6C). These results suggest that BK-induced TRPM8 inhibition is solely due to direct $G\alpha_q$ gating, consistent with the aforementioned finding in sensory DRG neurons lacking $G\alpha_q$ (Figures 2D–2G). This conclusion is also supported by the previous pharmacological evidence showing that inhibition of PLCβ by U73122 had no effect on BK-induced TRPM8 inhibition (Zhang et al., 2012), arguing against a possible role for PIP_2 hydrolysis in the functional coupling between B2R and TRPM8.

Potent inhibition of TRPM8 was also observed in cells co-expressing TRPM8 and H1R upon stimulation with histamine (Figure 6D). In this case, 93.5% of the peak inward current and 41% of the outward current of TRPM8 were inhibited by histamine, in agreement with the previous results (Zhang et al., 2012). Similar to BK treatment, TRPM8-TM abolished inhibition of outward current caused by histamine and significantly reduced histamine-induced inhibition of inward current, though histamine still inhibited 43% of the inward current of TRPM8-TM (Figures 6D and 6E). The remaining inhibition is likely contributed by PIP_2

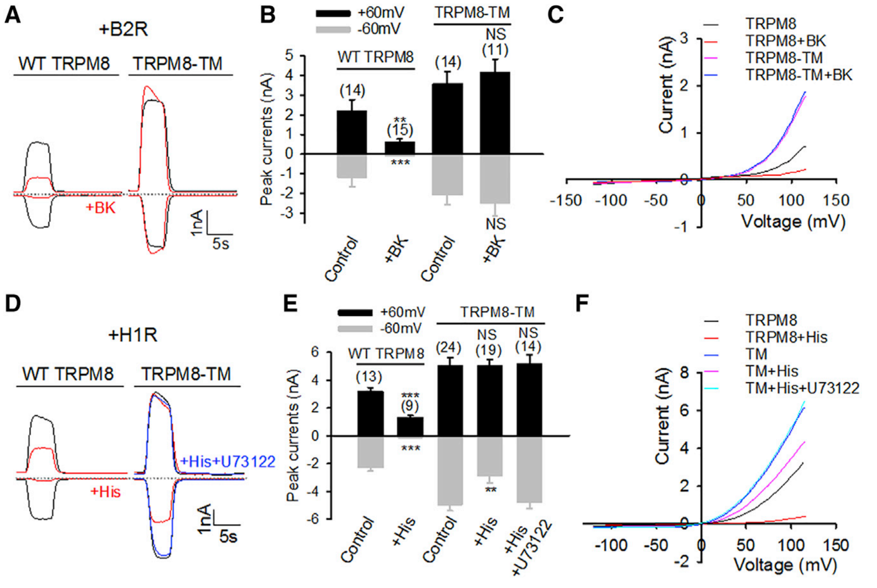

Figure 6. $G\alpha_q$ Gating Sites Are Essential for TRPM8 Inhibition by Inflammatory Mediators

(A) Example inward and outward currents elicited by menthol (200 μM, 5 s) in HEK293 cells co-expressing B2R and TRPM8 or TRPM8-TM without or with BK pretreatment (1 μM, 1 min, red).

(B) Summary of results similar to those in (A).

(C) Average I-V relationship of TRPM8 and TRPM8-TM with or without BK treatment (n = 7–13) from experiments similar to those in (A).

(D) Findings from experiments similar to those in (A) but from cells co-expressing H1R. Some cells were pretreated with histamine (His, 10 μM, 1 min, red) or together with U73122 (2.5 μM, blue).

(E) Summary of results similar to those in (D).

(F) Average I-V relationship of TRPM8 and TRPM8-TM with or without histamine and U73122 treatment (n = 6–16) from experiments similar to those in (D).

All data indicate mean ± SEM. $^{**}p < 0.01$; $^{***}p < 0.001$; NS, not significant. See also Figure S3.

hydrolysis due to actions of activated PLCβ. Indeed, the previous pharmacological experiments showed that inhibition of PLCβ by U73122 partially alleviated TRPM8 inhibition by histamine, though it had no effect on BK-induced TRPM8 inhibition (Zhang et al., 2012). To further corroborate this idea, cells expressing TRPM8-TM lacking $G\alpha_q$ gating were also pretreated with U73122 for simultaneous prevention of PIP_2 signaling. As expected, the inhibitory effect of histamine was completely abolished under this condition (Figures 6D and 6E).

Similar findings were also observed when TRPM8 was activated by membrane voltage. Figure 6F shows that inhibition of depolarization-induced outward current by histamine was completely abolished only by TRPM8-TM together with U73122 pretreatment, not by either alone. These data conclusively demonstrate that both direct $G\alpha_q$ gating and downstream PIP_2 hydrolysis contribute to histamine-evoked TRPM8 inhibition, with $G\alpha_q$ gating playing a primary role (~57%), and the involvement of other mechanisms is unlikely.

Taken together, direct $G\alpha_q$ gating of TRPM8 channels is the sole mechanism for BK-induced inhibition of TRPM8, without a significant role for PIP_2 hydrolysis. However, both $G\alpha_q$ gating and PIP_2 signaling contribute to histamine-triggered TRPM8 inhibition.

TRPM8 Is Insensitive to PIP_2 Depletion in the Presence of B2R

Both B2R and H1R are $G\alpha_q$-coupled GCPRs. How can PIP_2 signaling play a role in the modulation of TRPM8 initiated by H1R, but not by B2R, though both receptors use $G\alpha_q$ gating for modulation of TRPM8? To elucidate this question, I used the inducible PIP_2 depletion system in which 5-phosphatase (5-ptase) coupled to FK506-binding protein 12 (FKBP12) is rapidly recruited to the membrane through dimerization with a membrane-targeted fragment of mammalian target of rapamycin (mTOR) by rapamycin, resulting in the depletion of membrane PIP_2 (Varnai et al., 2006). I first confirmed that the addition of rapamycin elicited a rapid reduction of membrane PIP_2 with this system, as indicated by a prompt translocation of co-expressed Tubby-R332H-YFP, a reporter of membrane PIP_2 level, to the cytoplasm (Figures 7A and 7B). I then investigated the effect of PIP_2 depletion on TRPM8 currents in HEK293 cells expressing the inducible PIP_2-depleting system. As anticipated, TRPM8 currents were markedly inhibited by rapamycin (Figures 7C and 7D). A similar extent of inhibition by rapamycin was also observed with TRPM8-TM, suggesting that mutating $G\alpha_q$ gating sites does not affect the sensitivity of TRPM8 to PIP_2. However, strikingly, depletion of PIP_2 with rapamycin ceased to inhibit either TRPM8 or TRPM8-TM when B2R was co-expressed (Figures 7C and 7D). Co-expressed B2R also significantly inhibited the basal currents of TRPM8 and TRPM8-TM, likely due to reduced sensitivity of the channels to basal membrane PIP_2. In contrast, co-expression of H1R did not affect the responses of TRPM8 to PIP_2 depletion (Figure S4), suggesting that B2R, but not H1R, alters the sensitivity of TRPM8 channels to PIP_2. Consistent with this idea, TRPM8 binds to B2R, but not to H1R (Figure 7E). These results suggest that the binding of B2R to TRPM8 influences the PIP_2 sensitivity of TRPM8 channels, leading to lack of response of TRPM8 to PIP_2 depletion. These data also confirm the finding that direct $G\alpha_q$ gating is the sole mechanism of TRPM8 inhibition by BK, while histamine-induced TRPM8 inhibition involves both $G\alpha_q$ gating and PIP_2 signaling.

$G\alpha_q$ engages and activates PLCβ through extensive contacts with the catalytic domain in PLCβ via Switch regions and the α3 helix in $G\alpha_q$ (Waldo et al., 2010), the same regions also binding to the N and C termini of TRPM8 (Figure 3A). Notably, a cluster of acidic residues in Switch III of $G\alpha_q$ crucial for TRPM8 gating was also found critical to PLCβ binding and activation (Venkatakrishnan and Exton, 1996), raising the possibility that TRPM8 competes with PLCβ for $G\alpha_q$ binding, thereby interfering with $G\alpha_q$-PLCβ interaction and coupling. Indeed, TRPM8 prevented

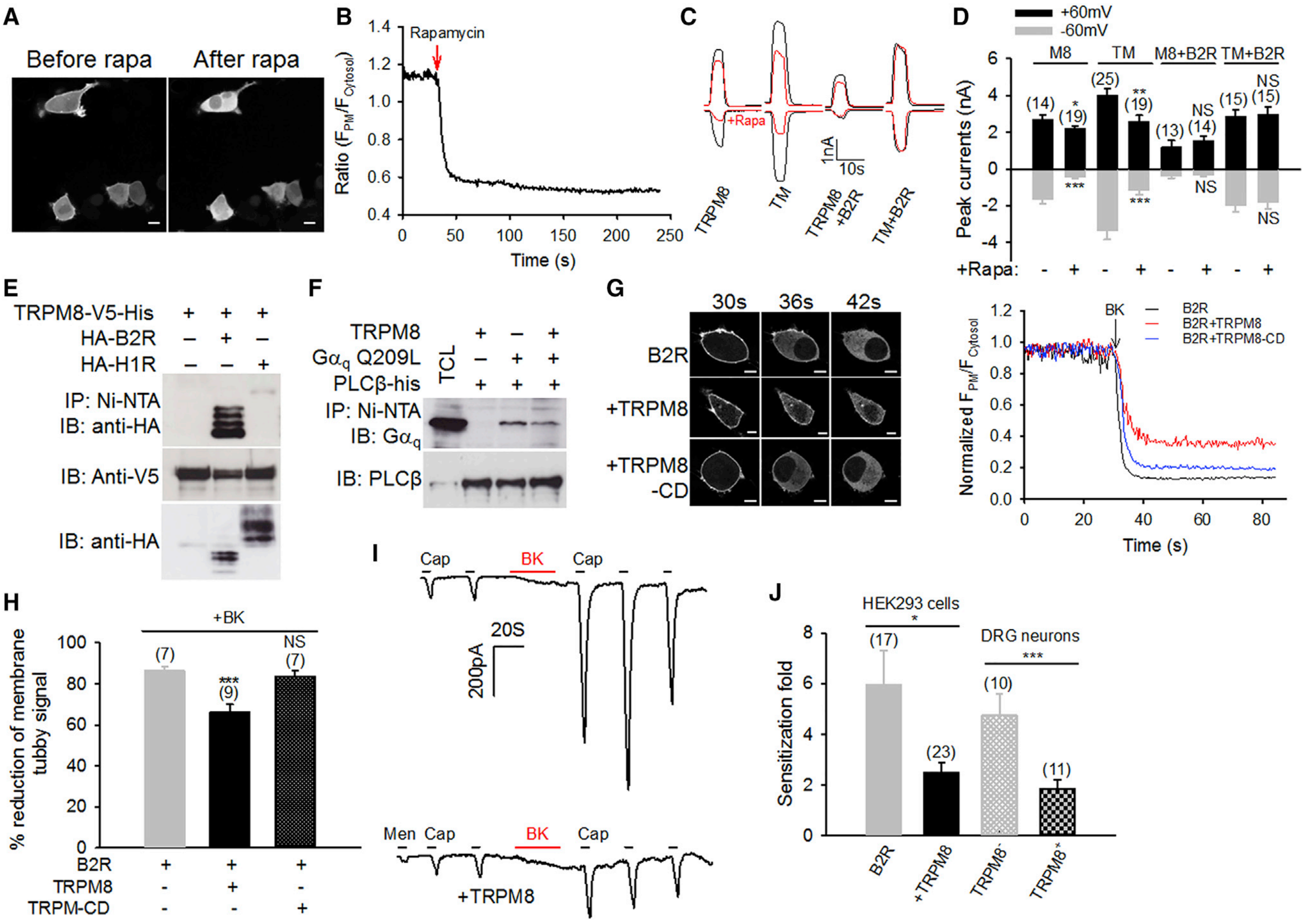

Figure 7. TRPM8 Is Insensitive to PIP_2 Depletion in the Presence of B2R

(A) Translocation of Tubby-R332H-cYFP induced by rapamycin (1 μM) in HEK293 cells co-expressing mRFP-FKBP-5-ptase domain and PM-FRB-CFP. Scale bars, 10 μM.

(B) Real-time quantification of membrane fluorescence (F_{PM}) relative to cytosol fluorescence ($F_{Cytosol}$) in the top left cell from (A).

(C) Example of whole-cell inward and outward currents elicited by menthol in HEK293 cells co-expressing mRFP-FKBP-5-ptase and PM-FRB-CFP together with either TRPM8 and B2R or TRPM8-TM and B2R. Red traces indicate cells pretreated with rapamycin (Rapa, 1 μM, 1 min).

(D) Collective results from experiments similar to those in (C).

(E) Binding of TRPM8 to B2R, but not to H1R, in a nickel-beads (Ni-NTA) pull-down assay performed on HEK293 cell lysate expressing the proteins as indicated.

(F) Binding between PLCβ-his (6×) and $G\alpha_q$ Q209L is reduced in the presence of TRPM8 in a nickel beads pull-down assay. Lane 1 indicates total cell lysate (TCL).

(G) Translocation of Tubby-R332H-cYFP at different time points after the addition of BK (1 μM) in HEK293 cells co-expressing B2R or together with TRPM8 or C-terminal deleted (TRPM8-CD). Scale bars, 10 μM. On the right, real-time quantification of membrane Tubby fluorescence is indicated relative to cytosol fluorescence from the cells in (G).

(H) Summary of results similar to those in (G).

(I) Example inward currents evoked by capsaicin (Cap, 100 nM, 5 s) in HEK293 cells expressing TRPV1 and B2R or together with TRPM8 before and after BK treatment (1 μM, red). TRPM8 co-expression is indicated by currents elicited by menthol (Men, 200 μM).

(J) Summary of TRPV1 sensitization fold induced by BK in experiments similar to those in (I). BK-induced sensitization of TRPV1 was also significantly different between $TRPM8^+$ and $TRPM8^-$ DRG neurons indicated by responses to menthol.

All error bars represent mean ± SEM. **p < 0.01; **p < 0.05; ***p < 0.001; NS, not significant. See also Figure S4.

the binding of $G\alpha_q$ to PLCβ in a nickel-beads pull-down assay (Figure 7F). Consistently, co-expression of TRPM8 also significantly reduced $G\alpha_q$-PLCβ signaling activated by BK, as indicated by a significantly smaller reduction of membrane Tubby-R332H-YFP fluorescence and, hence, less PIP_2 hydrolysis (Figures 7G and 7H). The reduced PIP_2 hydrolysis was, however, not observed in C-terminus-deleted TRPM8 (Figures 7G and 7H), supporting a role for the C terminus of TRPM8 in the intervention of $G\alpha_q$-PLCβ signaling. Functionally, co-expression of TRPM8 significantly reduced TRPV1 sensitization in both transfected HEK293 cells and DRG neurons induced by BK (Figures 7I and 7J), an event depending on $G\alpha_q$-PLCβ-PKC signaling (Zhang et al., 2008), further demonstrating that TRPM8 prevents $G\alpha_q$-PLC signaling.

In summary, B2R-$G\alpha_q$ and TRPM8 crosstalk, forming a bidirectional signaling complex. During $G\alpha_q$ gating of TRPM8,

TRPM8 binding also reciprocally interferes with $G\alpha_q$ binding and signaling to PLCβ and PIP_2. Concomitant B2R binding further renders TRPM8 insensitive to PIP_2. The crosstalk in the TRPM8-$G\alpha_q$-B2R signaling complex therefore fosters an outcome that direct $G\alpha_q$ gating—rather than PIP_2 signaling—is a sole mechanism for TRPM8 inhibition triggered by BK during inflammation.

DISCUSSION

In this study, I have investigated the gating mechanisms of TRPM8 by $G\alpha_q$ and its role in the modulation of the cold sensitivity of TRPM8 during inflammatory signaling. I found that $G\alpha_q$ constitutively binds to and gates TRPM8, inhibiting the basal cold sensitivity of TRPM8 in sensory DRG neurons under resting conditions. Direct $G\alpha_q$ gating is also responsible for TRPM8 inhibition elicited by BK and histamine under inflammatory conditions.

I further elucidated the mechanism of lack of a role for PIP_2 in the modulation of TRPM8 by BK. Surprisingly, B2R binds to TRPM8, rendering the channel insensitive to PIP_2 depletion. Meanwhile, TRPM8-$G\alpha_q$ binding interferes with $G\alpha_q$-PLCβ binding and signaling, resulting in diminished sensitization of TRPV1. B2R-$G\alpha_q$ and TRPM8, therefore, form a bidirectional signaling complex: on the one hand, $G\alpha_q$ and B2R engage TRPM8, leading to direct $G\alpha_q$ gating and diminished PIP_2 sensitivity of TRPM8, respectively; on the other hand, TRPM8 binding reciprocally prevents $G\alpha_q$-PLCβ binding and signaling, leading to reduced PIP_2 hydrolysis. The bidirectional signaling crosstalk in the B2R-$G\alpha_q$-TRPM8 complex, therefore, collectively determines direct $G\alpha_q$ gating as a sole means to inhibit TRPM8 by BK. Furthermore, inhibition of sensitization of TRPV1 by co-expressed TRPM8 suggests that a non-channel-function-dependent mechanism also contributes to the analgesic effect of TRPM8. In contrast to B2R, H1R neither binds to TRPM8 nor influences the PIP_2 sensitivity of TRPM8, consistent with the finding that PLCβ-PIP_2 signaling plays a role in histamine-evoked TRPM8 inhibition. These results suggest that the PIP_2 sensitivity of TRPM8 varies across DRG neurons influenced by co-expressed B2R.

I also revealed the molecular details of TRPM8-$G\alpha_q$ interaction and the functions of different binding regions. The present results support the hypothesis that the N terminus of TRPM8 is crucial to $G\alpha_q$ gating of TRPM8 through binding to Switch III in $G\alpha_q$, while the proximal C terminus interferes with $G\alpha_q$-PLCβ-PIP_2 signaling through binding to the α3 helix in $G\alpha_q$ (Figure 3A). The proximal C terminus contains the TRP domain that is critical to the binding and sensitivity of TRPM8 to PIP_2 (Rohács et al., 2005). $G\alpha_q$ binding to the TRP domain may, thus, also influence TRPM8-PIP_2 binding and the resultant TRPM8 sensitivity to PIP_2, in addition to diminished PLCβ-PIP_2 signaling. It is, therefore, likely that $G\alpha_q$ gating and PIP_2 signaling also crosstalk and cooperate to modulate TRPM8, in addition to their separate and independent roles in the modulation of TRPM8.

Apart from BK and histamine, my previous research suggests that activation of two other $G\alpha_q$-coupled GPCRs— the muscarinic receptor M1R and the chloroquine receptor MrgprA3—also inhibit TRPM8 through direct $G\alpha_q$ gating (Li and Zhang, 2013; Than et al., 2013). Interestingly, pharmacological inhibition of PLCβ with U73122 did not exert any effect on TRPM8 inhibition induced by the activation of either of these two GPCRs in a manner similar to that of B2R, suggesting that direct $G\alpha_q$ gating, but not PLCβ-PIP_2 signaling, is involved in the functional coupling of TRPM8 to both M1R and MrgprA3. Direct $G\alpha_q$ gating may be a primary mechanism underlying TRPM8 modulation by the broad family of $G\alpha_q$-coupled GPCRs.

This study also provided important insights into the mechanisms of $G\alpha_q$ gating of TRPM8 channels. I found that $G\alpha_q$ gates TRPM8 through electrostatic interactions with three basic arginine residues on the N-terminal MHR1–3 of TRPM8. Neutralization of these residues abrogated $G\alpha_q$ binding and its inhibitory effect on TRPM8. The mutation also dramatically enhanced TRPM8 sensitivity and shifted voltage gating close to resting membrane potential, explaining the enhanced cold sensitivity and cold-evoked firing response in DRG neurons lacking $G\alpha_q$. These unveiled $G\alpha_q$ gating sites provide insights into the general gating mechanisms of TRPM8 channels and will advance our understanding of TRPM8 modulation in physiology and diseases. They also suggest a critical role for the MHR domain in the gating of TRPM8 and, potentially, other members of the TRPM family.

How does $G\alpha_q$ gate TRPM8 channels? Our results suggest that R470 and the TRP domain in TRPM8 contact with Switch III and the α3 helix in $G\alpha_q$, respectively (Figure 3A). This notion is further supported by structural measurements showing that the distance between R470 and the TRP domain (~36 Å) matches the length of the α3 helix of $G\alpha_q$ (~30 Å) (Figures 4A and 5I). Structural modeling of TRPM8-$G\alpha_q$ interaction also suggests that R470 is close to E245 on $G\alpha_q$ and that the top of the α3 helix is close to the bottom of the TRP domain (<10 Å) (Figure S2). However, R364 and R368 near the bottom of the cavity are far from R470 (~47 Å) (Figure 5I). Thus, it seems impracticable that Switch III binds simultaneously to both R470 and R364-R368. However, structural modeling suggests that R364-R368 are close to the helical domain of $G\alpha_q$ (Figure S2), which also exhibits rich negative charges (Figure 4A), allowing for electrostatic interaction. This interaction may be responsible for the residual binding between the N terminus of TRPM8 and $5G\alpha_{qi}$ (Figure 3B). Overall, $G\alpha_q$ is likely tethered to the cytoplasmic cavity of TRPM8 through interactions with the top TRP domain, lateral R470, and bottom R364-R368 in the cytoplasmic cavity via the α3 helix, Switch III, and the helical domain, respectively (Figure S2). These interactions likely impede structural coupling between different TRPM8 domains, leading to allosteric inhibition of TRPM8 gating. Another prominent feature of the model is that the top of $G\alpha_q$ is close to S6 in addition to the TRP domain of TRPM8. Conformation changes during $G\alpha_q$ activation may, thus, allow $G\alpha_q$ to interact with both the TRP and pore domains, which are critical for integrating allosteric gating signals from cold, cooling agonists, voltage, and PIP_2 (Yin et al., 2019), mediating the allosteric gating effects of $G\alpha_q$. As Switch III in $G\alpha_q$ is one of the regions undergoing major conformational changes upon $G\alpha_q$ activation and accounts for the majority of binding to TRPM8, while little structural changes occurs to the α3 helix and the helical domain, it is likely that R470 acts as a major $G\alpha_q$ gating site, while R364-R368 function as ancillary tethering sites assisting in $G\alpha_q$ gating. However, the exact mechanisms of

TRPM8 gating by $G\alpha_q$ await future determination of the structure of the TRPM8-$G\alpha_q$ complex.

TRPM8 has been implicated in a plethora of pathological conditions and diseases, including pain (Colburn et al., 2007; De Caro et al., 2018; Dhaka et al., 2007; Knowlton et al., 2013; Liu et al., 2013; Proudfoot et al., 2006), inflammation (Caceres et al., 2017; Ramachandran et al., 2013; Wang et al., 2017), itch (Palkar et al., 2018), thermoregulation and energy metabolism (Li et al., 2018; Reimúndez et al., 2018), cancer (Yee, 2015), dry eye diseases (Yang et al., 2018), and airway diseases (Liu et al., 2018). TRPM8 has thus become an attractive therapeutic target for treating these conditions and diseases. Potent inhibition of TRPM8 by $G\alpha_q$-coupled GPCRs provides additional therapeutic possibilities for alleviating these diseases and conditions, for example, through existing GPCR agonists or antagonists for modulating TRPM8 activities in affected tissues and organs. Furthermore, in terms of the marked impact of the $G\alpha_q$ effector sites on TRPM8 gating, $G\alpha_q$ gating sites will constitute excellent targets for high-fidelity drug discovery for effective manipulation of TRPM8 function.

Altogether, the discovery of a prominent gating mechanism of TRPM8 by $G\alpha_q$ and its application to inflammation in this research will advance the understanding of the role of TRPM8 in physiology and diseases and provide the basis and guidance for developing intervention strategies for combating TRPM8-associated diseases.

STAR★METHODS

Detailed methods are provided in the online version of this paper and include the following:

- KEY RESOURCES TABLE
- LEAD CONTACT AND MATERIALS AVAILABILITY
- EXPERIMENTAL MODEL AND SUBJECT DETAILS
  - Animals
  - DRG neurons and cell lines
- METHOD DETAILS
  - Cell transfection
  - Molecular Biology
  - Pull down assay and western blot
  - Electrophysiology
  - Fluorescence imaging
  - Protein structure modeling
- QUANTIFICATION AND STATISTICAL ANALYSIS

SUPPLEMENTAL INFORMATION

Supplemental Information can be found online at https://doi.org/10.1016/j.celrep.2019.05.080.

ACKNOWLEDGMENTS

I would like to thank Dr. Dan Rathbone for assistance with protein structure modeling and Dr. Gerry Hammond for help with live imaging and analysis. This work was supported by an MRC grant (G0801387) and intramural funding from Aston University. Original data for this work are available through Aston data Explorer at https://doi.org/10.17036/researchdata.aston.ac.uk.00000427.

AUTHOR CONTRIBUTIONS

X.Z. designed and performed experiments, analyzed data, and wrote the paper.

DECLARATION OF INTERESTS

The author declares no competing interests.

Received: January 12, 2019
Revised: April 3, 2019
Accepted: May 21, 2019
Published: June 18, 2019

REFERENCES

Bautista, D.M., Siemens, J., Glazer, J.M., Tsuruda, P.R., Basbaum, A.I., Stucky, C.L., Jordt, S.E., and Julius, D. (2007). The menthol receptor TRPM8 is the principal detector of environmental cold. Nature *448*, 204–208.

Caceres, A.I., Liu, B., Jabba, S.V., Achanta, S., Morris, J.B., and Jordt, S.E. (2017). Transient receptor potential cation channel subfamily M member 8 channels mediate the anti-inflammatory effects of eucalyptol. Br. J. Pharmacol. *174*, 867–879.

Colburn, R.W., Lubin, M.L., Stone, D.J., Jr., Wang, Y., Lawrence, D., D'Andrea, M.R., Brandt, M.R., Liu, Y., Flores, C.M., and Qin, N. (2007). Attenuated cold sensitivity in TRPM8 null mice. Neuron *54*, 379–386.

Daniels, R.L., Takashima, Y., and McKemy, D.D. (2009). Activity of the neuronal cold sensor TRPM8 is regulated by phospholipase C via the phospholipid phosphoinositol 4,5-bisphosphate. J. Biol. Chem. *284*, 1570–1582.

De Caro, C., Russo, R., Avagliano, C., Cristiano, C., Calignano, A., Aramini, A., Bianchini, G., Allegretti, M., and Brandolini, L. (2018). Antinociceptive effect of two novel transient receptor potential melastatin 8 antagonists in acute and chronic pain models in rat. Br. J. Pharmacol. *175*, 1691–1706.

Dhaka, A., Murray, A.N., Mathur, J., Earley, T.J., Petrus, M.J., and Patapoutian, A. (2007). TRPM8 is required for cold sensation in mice. Neuron *54*, 371–378.

Dhaka, A., Earley, T.J., Watson, J., and Patapoutian, A. (2008). Visualizing cold spots: TRPM8-expressing sensory neurons and their projections. J. Neurosci. *28*, 566–575.

Gavva, N.R., Davis, C., Lehto, S.G., Rao, S., Wang, W., and Zhu, D.X. (2012). Transient receptor potential melastatin 8 (TRPM8) channels are involved in body temperature regulation. Mol. Pain *8*, 36.

Han, S.K., Mancino, V., and Simon, M.I. (2006). Phospholipase Cbeta 3 mediates the scratching response activated by the histamine H1 receptor on C-fiber nociceptive neurons. Neuron *52*, 691–703.

Hasan, R., Leeson-Payne, A.T., Jaggar, J.H., and Zhang, X. (2017). Calmodulin is responsible for Ca^{2+}-dependent regulation of TRPA1 Channels. Sci. Rep. *7*, 45098.

Knowlton, W.M., Daniels, R.L., Palkar, R., McCoy, D.D., and McKemy, D.D. (2011). Pharmacological blockade of TRPM8 ion channels alters cold and cold pain responses in mice. PLoS ONE *6*, e25894.

Knowlton, W.M., Palkar, R., Lippoldt, E.K., McCoy, D.D., Baluch, F., Chen, J., and McKemy, D.D. (2013). A sensory-labeled line for cold: TRPM8-expressing sensory neurons define the cellular basis for cold, cold pain, and cooling-mediated analgesia. J. Neurosci. *33*, 2837–2848.

Lambright, D.G., Sondek, J., Bohm, A., Skiba, N.P., Hamm, H.E., and Sigler, P.B. (1996). The 2.0 A crystal structure of a heterotrimeric G protein. Nature *379*, 311–319.

Li, L., and Zhang, X. (2013). Differential inhibition of the TRPM8 ion channel by Gαq and Gα 11. Channels (Austin) *7*, 115–118.

Li, C., Li, J., Xiong, X., Liu, Y., Lv, Y., Qin, S., Liu, D., Wei, R., Ruan, X., Zhang, J., et al. (2018). TRPM8 activation improves energy expenditure in skeletal muscle and exercise endurance in mice. Gene *641*, 111–116.

Linte, R.M., Ciobanu, C., Reid, G., and Babes, A. (2007). Desensitization of cold- and menthol-sensitive rat dorsal root ganglion neurones by inflammatory mediators. Exp. Brain Res. *178*, 89–98.

Liu, B., and Qin, F. (2005). Functional control of cold- and menthol-sensitive TRPM8 ion channels by phosphatidylinositol 4,5-bisphosphate. J. Neurosci. *25*, 1674–1681.

Liu, B., Fan, L., Balakrishna, S., Sui, A., Morris, J.B., and Jordt, S.E. (2013). TRPM8 is the principal mediator of menthol-induced analgesia of acute and inflammatory pain. Pain *154*, 2169–2177.

Liu, H., Liu, Q., Hua, L., and Pan, J. (2018). Inhibition of transient receptor potential melastatin 8 alleviates airway inflammation and remodeling in a murine model of asthma with cold air stimulus. Acta Biochim. Biophys. Sin. (Shanghai) *50*, 499–506.

Lukacs, V., Thyagarajan, B., Varnai, P., Balla, A., Balla, T., and Rohacs, T. (2007). Dual regulation of TRPV1 by phosphoinositides. J. Neurosci. *27*, 7070–7080.

Madrid, R., Donovan-Rodríguez, T., Meseguer, V., Acosta, M.C., Belmonte, C., and Viana, F. (2006). Contribution of TRPM8 channels to cold transduction in primary sensory neurons and peripheral nerve terminals. J. Neurosci. *26*, 12512–12525.

Madrid, R., de la Peña, E., Donovan-Rodriguez, T., Belmonte, C., and Viana, F. (2009). Variable threshold of trigeminal cold-thermosensitive neurons is determined by a balance between TRPM8 and Kv1 potassium channels. J. Neurosci. *29*, 3120–3131.

McKemy, D.D., Neuhausser, W.M., and Julius, D. (2002). Identification of a cold receptor reveals a general role for TRP channels in thermosensation. Nature *416*, 52–58.

Offermanns, S., Hashimoto, K., Watanabe, M., Sun, W., Kurihara, H., Thompson, R.F., Inoue, Y., Kano, M., and Simon, M.I. (1997a). Impaired motor coordination and persistent multiple climbing fiber innervation of cerebellar Purkinje cells in mice lacking Galphaq. Proc. Natl. Acad. Sci. USA *94*, 14089–14094.

Offermanns, S., Toombs, C.F., Hu, Y.H., and Simon, M.I. (1997b). Defective platelet activation in G alpha(q)-deficient mice. Nature *389*, 183–186.

Palkar, R., Ongun, S., Catich, E., Li, N., Borad, N., Sarkisian, A., and McKemy, D.D. (2018). Cooling relief of acute and chronic itch requires TRPM8 channels and neurons. J. Invest. Dermatol. *138*, 1391–1399.

Parra, A., Madrid, R., Echevarria, D., del Olmo, S., Morenilla-Palao, C., Acosta, M.C., Gallar, J., Dhaka, A., Viana, F., and Belmonte, C. (2010). Ocular surface wetness is regulated by TRPM8-dependent cold thermoreceptors of the cornea. Nat. Med. *16*, 1396–1399.

Peier, A.M., Moqrich, A., Hergarden, A.C., Reeve, A.J., Andersson, D.A., Story, G.M., Earley, T.J., Dragoni, I., McIntyre, P., Bevan, S., and Patapoutian, A. (2002). A TRP channel that senses cold stimuli and menthol. Cell *108*, 705–715.

Perkins, K.L. (2006). Cell-attached voltage-clamp and current-clamp recording and stimulation techniques in brain slices. J. Neurosci. Methods *154*, 1–18.

Premkumar, L.S., Raisinghani, M., Pingle, S.C., Long, C., and Pimentel, F. (2005). Downregulation of transient receptor potential melastatin 8 by protein kinase C-mediated dephosphorylation. J. Neurosci. *25*, 11322–11329.

Proudfoot, C.J., Garry, E.M., Cottrell, D.F., Rosie, R., Anderson, H., Robertson, D.C., Fleetwood-Walker, S.M., and Mitchell, R. (2006). Analgesia mediated by the TRPM8 cold receptor in chronic neuropathic pain. Curr. Biol. *16*, 1591–1605.

Quinn, K.V., Behe, P., and Tinker, A. (2008). Monitoring changes in membrane phosphatidylinositol 4,5-bisphosphate in living cells using a domain from the transcription factor tubby. J Physiol *586*, 2855–2871.

Ramachandran, R., Hyun, E., Zhao, L., Lapointe, T.K., Chapman, K., Hirota, C.L., Ghosh, S., McKemy, D.D., Vergnolle, N., Beck, P.L., et al. (2013). TRPM8 activation attenuates inflammatory responses in mouse models of colitis. Proc. Natl. Acad. Sci. USA *110*, 7476–7481.

Reimúndez, A., Fernández-Peña, C., García, G., Fernández, R., Ordás, P., Gallego, R., Pardo-Vazquez, J.L., Arce, V., Viana, F., and Señarís, R. (2018). Deletion of the cold thermoreceptor TRPM8 increases heat loss and food intake leading to reduced body temperature and obesity in mice. J. Neurosci. *38*, 3643–3656.

Rohács, T., Lopes, C.M., Michailidis, I., and Logothetis, D.E. (2005). PI(4,5)P2 regulates the activation and desensitization of TRPM8 channels through the TRP domain. Nat. Neurosci. *8*, 626–634.

Than, J.Y., Li, L., Hasan, R., and Zhang, X. (2013). Excitation and modulation of TRPA1, TRPV1, and TRPM8 channel-expressing sensory neurons by the pruritogen chloroquine. J. Biol. Chem. *288*, 12818–12827.

Varnai, P., Thyagarajan, B., Rohacs, T., and Balla, T. (2006). Rapidly inducible changes in phosphatidylinositol 4,5-bisphosphate levels influence multiple regulatory functions of the lipid in intact living cells. J. Cell Biol. *175*, 377–382.

Venkatakrishnan, G., and Exton, J.H. (1996). Identification of determinants in the alpha-subunit of Gq required for phospholipase C activation. J. Biol. Chem. *271*, 5066–5072.

Waldo, G.L., Ricks, T.K., Hicks, S.N., Cheever, M.L., Kawano, T., Tsuboi, K., Wang, X., Montell, C., Kozasa, T., Sondek, J., and Harden, T.K. (2010). Kinetic scaffolding mediated by a phospholipase C-beta and Gq signaling complex. Science *330*, 974–980.

Wang, X.P., Yu, X., Yan, X.J., Lei, F., Chai, Y.S., Jiang, J.F., Yuan, Z.Y., Xing, D.M., and Du, L.J. (2017). TRPM8 in the negative regulation of TNFα expression during cold stress. Sci. Rep. *7*, 45155.

Yang, J.M., Wei, E.T., Kim, S.J., and Yoon, K.C. (2018). TRPM8 channels and dry eye. Pharmaceuticals (Basel) *11*, E125.

Yee, N.S. (2015). Roles of TRPM8 ion channels in cancer: proliferation, survival, and invasion. Cancers (Basel) *7*, 2134–2146.

Yin, Y., Wu, M., Zubcevic, L., Borschel, W.F., Lander, G.C., and Lee, S.Y. (2018). Structure of the cold- and menthol-sensing ion channel TRPM8. Science *359*, 237–241.

Yin, Y., Le, S.C., Hsu, A.L., Borgnia, M.J., Yang, H., and Lee, S.Y. (2019). Structural basis of cooling agent and lipid sensing by the cold-activated TRPM8 channel. Science *363*, eaav9334.

Zakharian, E., Thyagarajan, B., French, R.J., Pavlov, E., and Rohacs, T. (2009). Inorganic polyphosphate modulates TRPM8 channels. PLoS ONE *4*, e5404.

Zhang, X., Li, L., and McNaughton, P.A. (2008). Proinflammatory mediators modulate the heat-activated ion channel TRPV1 via the scaffolding protein AKAP79/150. Neuron *59*, 450–461.

Zhang, X., Mak, S., Li, L., Parra, A., Denlinger, B., Belmonte, C., and McNaughton, P.A. (2012). Direct inhibition of the cold-activated TRPM8 ion channel by Gαq. Nat. Cell Biol. *14*, 851–858.

Zywietz, A., Gohla, A., Schmelz, M., Schultz, G., and Offermanns, S. (2001). Pleiotropic effects of Pasteurella multocida toxin are mediated by Gq-dependent and -independent mechanisms. involvement of Gq but not G11. J. Biol. Chem *276*, 3840–3845.

STAR★METHODS

KEY RESOURCES TABLE

REAGENT or RESOURCE	SOURCE	IDENTIFIER
Antibodies		
Mouse monoclonal anti-HA.11	Covance	Cat# MMS-101R-1000; RRID:AB_291262
Mouse monoclonal anti-β-Tubulin	Sigma-Aldrich	Cat# T4026; RRID:AB_477577
Rabbit polyclonal anti-Gaq	Santa Cruz	Cat# sc-393; RRID:AB_631536
Rabbit polyclonal anti-Ga11	Santa Cruz	Cat# sc-394; RRID:AB_2111195
Rabbit polyclonal anti-PLCβ	Santa Cruz	Cat# sc-9050; RRID:AB_2165496
Rabbit polyclonal anti-TRPM8	Alomone labs	Cat# ACC-049; RRID:AB_2040254
Anti-GST HRP conjugated	GE healthcare	Cat# RPN1236; RRID:AB_771429
Mouse monoclonal anti-V5 tag	Thermo Fisher	Cat# R960-25; RRID:AB_2556564
ECL HRP-linked rabbit whole Ab	GE healthcare	Cat# NA934; RRID:AB_772206
ECL HRP-linked mouse whole Ab	GE healthcare	Cat# NA931; RRID:AB_772210
Biological Samples		
Mice dorsal root ganglia	This paper	N/A
Chemicals, Peptides, and Recombinant Proteins		
DMEM media	Thermo Fisher	Cat# 11564446
Protease inhibitors	Roche Diagnostics	Cat# 11836170001
FBS	Thermo Fisher	Cat# 11550356
TurboFect reagent	Thermo Fisher	Cat# 15325016
Mouse Laminin	Becton Dickinson	Cat# 354232
NGF	Promega	Cat# G5141
Poly-L-lysine	Sigma-Aldrich	Cat# P9155
Cytosine β-D-arabinofuranoside	Sigma-Aldrich	Cat# C1768
Collagenase type IV	Worthington	Cat# LS004186
Bradykinin	Tocris Bioscience	Car# 3004
Rapamycin	Tocris Bioscience	Cat# 1292
Quick-change mutagenesis kit	Agilent Technologies	Cat# 200521
PBMC	Focus Biomolecules	Cat# 10-1413
Glutathione-agarose	Sigma-Aldrich	Cat# G4510
Experimental Models: Cell Lines		
HEK293T	ATCC	Cat# CRL11268
$G\alpha_q^{-/-}$ MEF cells	Provided by Prof. Stefan Offermanns	Zywietz et al., 2001
Experimental Models: Organisms/Strains		
$G\alpha_q$ knockout mice	Provided by Prof. Stefan Offermanns	Offermanns et al., 1997b
Recombinant DNA		
Rat TRPM8-V5-His	Zhang et al., 2012	N/A
G protein alpha q	cDNA resource center	Cat# GNA0Q00000
Bradykinin receptor B2R	cDNA resource center	Cat# BDKB20TN00
Histamine receptor H1R	cDNA resource center	Cat# HRH010TN00
6xHis-tag PLCβ1	Provided by Prof. Elliott M. Ross (UTSMC)	N/A

(*Continued on next page*)

Continued

REAGENT or RESOURCE	SOURCE	IDENTIFIER
mRFB-FKBP-5-ptase	Provided by Dr. Gerry Hammond (University of Pittsburgh)	Varnai et al., 2006
PM-FRB-CFP	Provided by Dr. Gerry Hammond	Varnai et al., 2006
Tubby-R332H-cYFP	Provided by Dr. Gerry Hammond	Quinn et al., 2008
GST-pcDNA3	Hasan et al., 2017	N/A
Software and Algorithms		
Sigmaplot	Systat Software Inc.	https://systatsoftware.com/products/sigmaplot/
pClamp11	Molecular Device	https://www.moleculardevices.com/products/axon-patch-clamp-system/acquisition-and-analysis-software/pclamp-software-suite
Discovery studio 4.5	BIOVIA	https://www.3dsbiovia.com/products/collaborative-science/biovia-discovery-studio/
ImageJ	NIH	https://imagej.nih.gov/ij/
Other		
Axopatch 200B amplifier	Molecular Devices	https://www.moleculardevices.com/products/axon-patch-clamp-system/amplifiers/axon-instruments-patch-clamp-amplifiers#gref
Confocal microscope	Leica	https://www.leica-microsystems.com/products/confocal-microscopes/

LEAD CONTACT AND MATERIALS AVAILABILITY

Further information and requests for resources and reagents should be directed to and will be fulfilled by the lead contact, Xuming Zhang (x.zhang39@aston.ac.uk).

EXPERIMENTAL MODEL AND SUBJECT DETAILS

Animals

$G\alpha_q^{-/-}$ mice were kindly provided by Prof. Stefan Offermanns (Max-Planck-Institute, Bad Nauheim, Germany). The male and female mice and their littermates were maintained on a C57BL/6 background housed in a 12h light/dark cycle with food and water *ad libitum.* The experiments on mice were approved by the ethical review committee of Aston University and UK home office and carried out in accordance with the Animal Scientific Procedures Act 1986 in the UK. The knockout mice are viable but exhibit impaired motor coordination and defective platelet activation (Offermanns et al., 1997a; Offermanns et al., 1997b).

DRG neurons and cell lines

DRG neurons were isolated from male and female wild-type or $G\alpha_q^{-/-}$ mice of 2-7 days after birth following sacrifice by cervical dislocation and cultured as described previously (Than et al., 2013; Zhang et al., 2008). Briefly, isolated DRGs were treated with type IV collagenase (Worthington, LS004186) followed by trituration. The dissociated DRG neurons were then seeded onto coverslips coated with poly-L-lysine (Sigma, P9155) and laminin (BD, 354232) and maintained in DMEM media (Thermo fisher, 11564446) containing 10% fetal bovine serum (Thermo Fisher, 11550356), 2mM L-glutamine, 100IU/ml penicillin, 100 μg/ml streptomycin supplemented with 5 μM cytosine β-D-arabinofuranoside (Sigma, C1768) and 50ng/ml nerve growth factor (Promega, G5141). The cultured DRG neurons were used within 24 hours after isolation.

HEK293 cell line was purchased from ATCC, and MEF cells derived from $G\alpha_{q/11}^{-/-}$ mice were obtained from Prof. Stefan Offermanns (Max-Planck-Institute, Germany) as described previously (Zhang et al., 2012). The cells lines were maintained in high glucose DMEM media containing 10% fetal bovine serum, 100IU/ml penicillin and 100 μg/ml streptomycin at 37°C in a humidified incubator (5% CO_2). Cells were used between 15 and 30 passage numbers. The cell lines were derived from embryos and the sex of these cell lines is thus not available.

METHOD DETAILS

Cell transfection

HEK293 and MEF cells were transfected with plasmid cDNAs using TurboFect transfection reagent (Thermo Fisher Scientific, 15325016) as described previously (Hasan et al., 2017; Zhang et al., 2012). Briefly, 4 μg cDNA in 400 μL serum-free media was mixed

with 8 μL TurboFect reagent followed by incubation for 20 min at RT. The transfection complexes were then added to the cells and incubated for 24h. For single cell electrophysiology, cells were co-transfected with GFP for identification of successfully transfected cells.

Molecular Biology

GST-coupled TRPM8 cytoplasmic fragments were constructed by amplifying relevant N- and C-terminal fragments from rTRPM8 with PCR followed by cloning into the GST-pCDNA3 vector via BamHI and EcoRI (Hasan et al., 2017). Truncation of GST-coupled protein fragments was produced by introducing a stop codon at targeted sites using Quick-Change mutagenesis kit (Agilent Technologies, 200521). The kit was also used to generate all the TRPM8 mutants. cNDAs for HA-B2R (BDKB20TN00) and HA-H1R (HRH010TN00) and $G\alpha_q$ (GNA0Q00000) were from the Missouri cDNA resource center. PLCβ-His (6x) cNDA is a kind gift from Dr. Elliott Ross (University of Texas Southwestern Medical Center at Dallas). The membrane lipid PIP_2 depletion was induced using mRFB-FKBP-5-ptase catalytic domain together with membrane targeted PM-FRB-CFP (kindly provide by Dr. Gerry Hammond, University of Pittsburgh), as described (Varnai et al., 2006). All other cDNA constructs including TRPM8-V5-His, Tubby-R332H-YFP and $G\alpha_{qi}$ chimeras have been described in the previous paper (Zhang et al., 2012).

Pull down assay and western blot

GST pull down assay was performed as described previously (Hasan et al., 2017; Zhang et al., 2012). Briefly, GST-coupled TRPM8 cytoplasmic fragments expressed from HEK293 cells were isolated using GST-agarose (Sigma, G4510), and then incubated with protein lysate containing $G\alpha_q$ Q209L in a binding buffer (20mM Tris-HCl, pH 7.4, 150mM NaCl, 1% NP-40, 1mM EDTA, 1mM EGTA and protease inhibitor cocktails (Roche, 11836170001)) at 4°C overnight. Bound $G\alpha_q$ proteins were eluted by boiling in sample buffer followed by separation in 10% SDS-PAGE gel and detection with primary anti-$G\alpha_q$ (Santa Cruz, sc-393) and secondary HRP-linked rabbit antibody (GE healthcare, NA934). The blots were stripped and redetected with HRP-conjugated anti-GST (GE healthcare, RPN1236).

Nickel beads pull down assay was conducted as described previously with slight modifications (Zhang et al., 2012). Briefly, HEK293 cells expressing TRPM8-V5-His with HA-B2R or with HA-H1R were solubilized in lysis buffer (20mM Tris-HCl, pH7.4, 300mM NaCl, 1% NP-40, 10% glycerol, 0.2mM EDTA, 20mM imidazole plus protease inhibitor cocktail). Protein lysates were then incubated with nickel beads (QIAGEN) at 4°C overnight. After thorough washing in lysis buffer, nickel beads were boiled in sample buffer. Eluted proteins were resolved on 7.5% SDS-PAGE gel followed by western blot detection with anti-HA (Covance, 101R-1000) and anti-V5 (Thermo Fisher, R960-25) together with secondary HRP-linked mouse antibody (GE healthcare, NA931). Similar nickel pull down assay was also performed for determination of the effect of TRPM8 on $G\alpha_q$ binding to hexahistidine-tagged PLCβ (Figure 7E). In this case, HEK293 cell lysate expressing TRPM8 was added to the cell lysate containing PLCβ-his and $G\alpha_q$ Q209L. After incubation at 4°C overnight, the cell lysate mixture was thoroughly washed and bound $G\alpha_q$ protein was then detected with anti-$G\alpha_q$ followed by stripping and redetection with anti-PLCβ (Santa Cruz, sc-9050). All the blots shown are representative of at least three independent experiments.

The expression level of TRPM8, $G\alpha_q$ and $G\alpha_{11}$ in $G\alpha_q^{-/-}$ mice was detected using western blot with anti-TRPM8 (Alomone labs, ACC-049), anti-$G\alpha_q$ (Santa Cruz, sc-393) and anti-$G\alpha_{11}$ (Santa Cruz, sc-394), respectively. The blots were also stripped and redetected with anti-β-tubulin (Sigma, T4026) for verifying equal loading across samples.

Electrophysiology

All electrophysiology recordings were obtained using an Axopatch 200B patch amplifier (Molecular Devices) as described previously (Hasan et al., 2017; Zhang et al., 2012). Briefly, patch pipettes were fabricated from thin-walled glass capillary using a pipette puller (Sutter instrument) with a resistance between 2.5 ~4.0 MΩ and filled with internal solution (in mM): 140 KCl, 2.0 MgCl2, 5 EGTA. 10 HEPES, pH 7.4 with KOH. Ca^{2+}-free extracellular solution was used and contained (in mM): 140 NaCl, 4 KCl, 10 HEPES, 1 MgCl2, 5 EGTA, 5 Glucose, pH7.4 with NaOH. Ca^{2+}-containing Hanks solution was largely similar to Ca^{2+}-free solution except that 5mM EGTA in Ca^{2+}-free solution was replaced with 2mM $CaCl_2$. For studying the effect of GPCR and PIP_2 signaling on TRPM8 in Figure 6 and 7, cells were pretreated with BK (Tocris, 3004) or histamine or rapamycin (Tocris, 1292) for 1min to allow intact completion of signaling before entering whole-cell configuration. After establishment of whole-cell, series resistance was 80% compensated. Signals were analog filtered at 1 KHz using a low-pass Bessel filter of the amplifier and digitized with Digidata 1440A (Molecular Devices). Recordings were made at RT with a holding potential at −60mV or + 60mV.

The current-voltage (I-V) relationship of TRPM8 was determined using a ramp depolarization from −120mV to +120mV in 650 msec. Voltage-dependent activation of TRPM8 was further investigated from currents evoked by depolarization voltage steps from −120mV up to +200mV in 20mV increments with every step lasting for 100msec.The half-maximal activation voltage ($V_{1/2}$) was calculated using the Boltzmann equation as described previously (Zhang et al., 2012).

Cell-attached recordings were performed as described previously (Madrid et al., 2006; Perkins, 2006; Zhang et al., 2012). Patch pipettes were pulled from thick-walled glass tubing with a resistance between 8 ~12 MΩ and a tight seal with seal resistance over 1GΩ was typical obtained. Cell-attached voltage-clamp mode was then used to measure action potential currents in DRG neurons evoked by different stimuli under normal Ca^{2+}-containing Hanks solution (see above). Cell-attached patch clamping maintains the intracellular contents of cells allowing for reliable and long lasting recording of action potentials. As TRPM8 is mainly expressed in

small-to-medium sized DRG neurons (Dhaka et al., 2008), we probed at random the responses of small-to-medium DRG neurons to cold, WS-12 and PBMC (Focus Biomolecules, 10-1413) for identification of $TRPM8^+$ neurons. Normal Hanks solution was used for both bath and pipette solutions.

Fluorescence imaging

Imaging of Tubby-R332H-YFP in live cells were performed using a Leica confocal microscope (Leica) as described previously (Li and Zhang, 2013; Than et al., 2013; Zhang et al., 2012). Fluorescence signals were acquired every 0.75 s. Bradykinin (1 μM) or rapamycin (1 μM) was added to the bath solution during imaging. Membrane PIP_2 depletion was estimated by quantifying the ratio of fluorescence signals on the membrane to those in the cytosol using ImageJ (NIH).

Protein structure modeling

The structure of TRPM8 (PDB: 6BPQ) and $G\alpha_q$ (PDB: 3OHM) was analyzed and modeled using Discovery studio 4.5 (BIOVIA).

QUANTIFICATION AND STATISTICAL ANALYSIS

Firing events of DRG neurons and TRPM8 currents were quantified and analyzed using the Clampfit 10.2 software (Molecular Devices). The number of cells was denoted in parentheses above the bars of each figure and derived from at least three different days of preparations. All the data passed the normality test and are shown as mean ± SEM. Significance between groups was determined using Student's t test or one-way ANOVA, and considered significant at $p < 0.05$.

Cell Reports, Volume *27*

Supplemental Information

Direct Gα_q Gating Is the Sole Mechanism for TRPM8 Inhibition Caused by Bradykinin Receptor Activation

Xuming Zhang

Supplemental Information

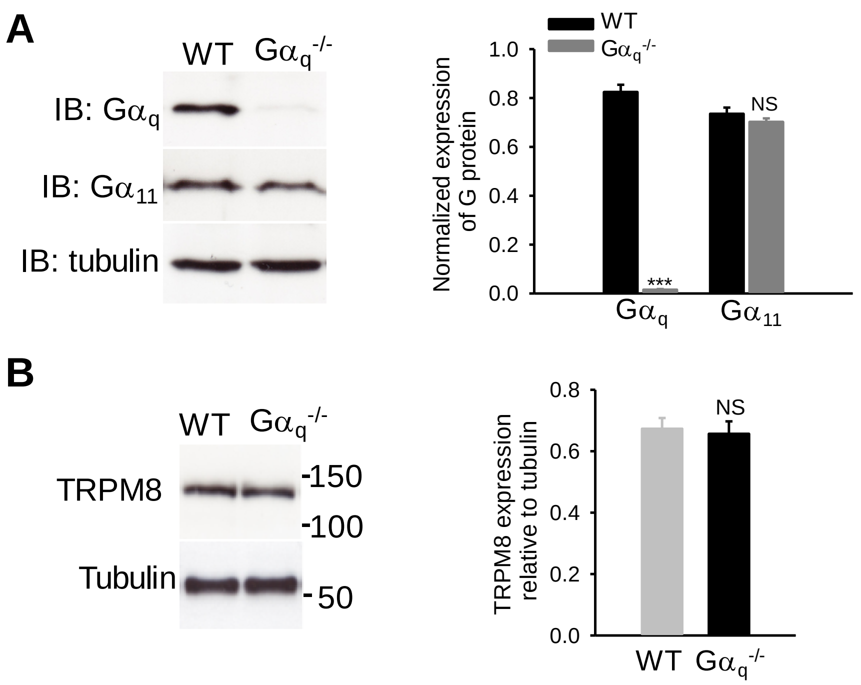

Figure S1. Gα_q is Deleted in DRG neurons from G$\alpha_q^{-/-}$ Mice (related to Figure 1).
(A) Expression of Gα_q and Gα_{11} in DRG neurons from wild-type (WT) and G$\alpha_q^{-/-}$ mice. On the right is the summary of expression of Gα_q and Gα_{11} relative to tubulin from experiments similar to those on the left. *** *P*<0.001; NS, not significant (n=3). **(B)** Example of TRPM8 expression in DRG neurons from WT and G$\alpha_q^{-/-}$ mice. On the right is the quantification of TRPM8 expression relative to that of β-tubulin from experiments similar to those on the left. NS, not significant (n=3).

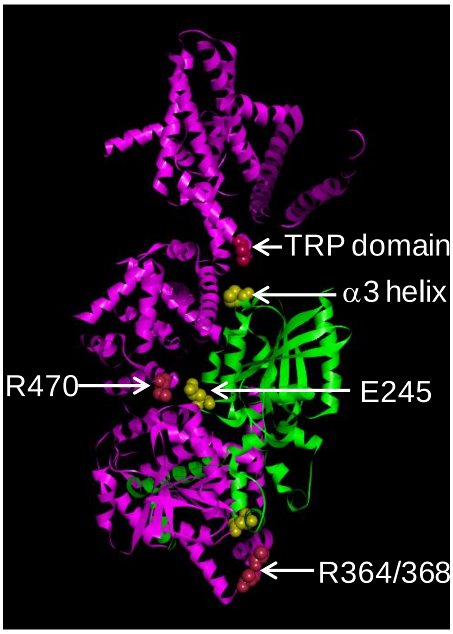

Figure S2. Ribbon Representation of Modelling Interactions between TRPM8 and Gα_q (related to Figure 5). The top TRP domain (V992), lateral R470 and bottom R364/R368 lining the cytoplasmic cavity of TRPM8 are in close proximity to α3 helix (P262), the Switch III domain (E245) and the helical domain in Gα_q (D138),

respectively. Potential interacting residues (R470-E245, R364/368-D138) between these domains are shown as balls.

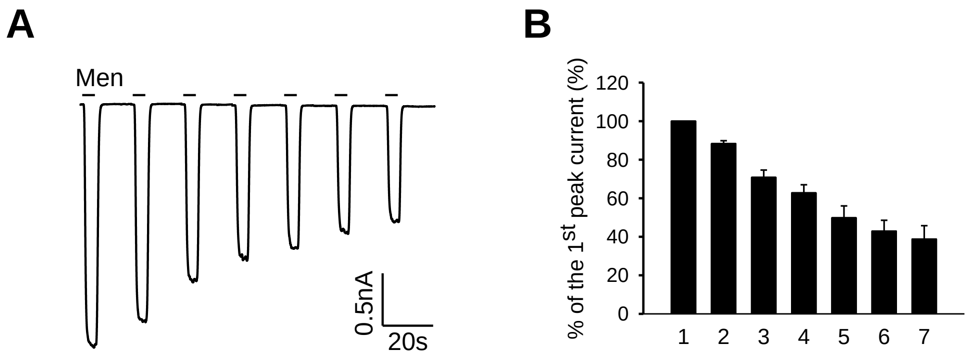

Figure S3. Run-down of TRPM8 Currents (related to Figure 6). (A) Example current traces in HEK293 cells expressing TRPM8 elicited by a series of menthol (200μM, 5s) in Ca^{2+}-free solution. **(B)** Summary of percentage of TRPM8 currents relative to the 1st peak current (n=5).

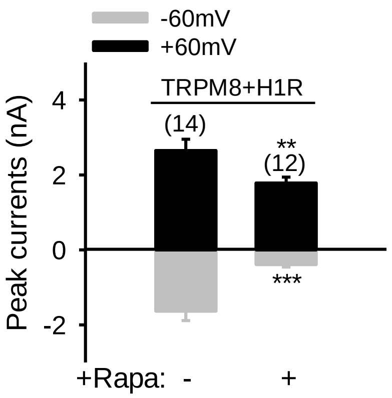

Figure S4. PIP_2 Depletion Inhibits TRPM8 in the Presence of H1R (related to Figure 7). Bar summary of peak inward and outward currents (-60mV and +60mV holding, respectively) of TRPM8 co-expressed with the inducible PIP_2 depletion system and H1R from experiments similar to those in Figure 7C. Depletion of PIP_2 by adding rapamycin (+) (Rapa, 1μM, 1min) inhibited TRPM8 currents. The number of experiments is shown above each bar. ***P* < 0.01; ****P* < 0.001.
